# Belastung von Eltern mit Kindern im Schulalter während verschiedener Phasen der COVID-19-Pandemie in Deutschland: Eine Analyse der COVID-19-Snapshot-Monitoring-(COSMO‑)Daten

**DOI:** 10.1007/s00103-021-03453-3

**Published:** 2021-11-26

**Authors:** Julia Elisabeth Rabe, Hannah Schillok, Christina Merkel, Stephan Voss, Michaela Coenen, Freia De Bock, Ursula von Rüden, Anke Bramesfeld, Caroline Jung-Sievers, COSMO-Gruppe, Cornelia Betsch, Lars Korn, Lisa Felgendreff, Sarah Eitze, Philipp Schmid, Philipp Sprengholz, Lothar Wieler, Patrick Schmich, Heidrun Thaiss, Freia De Bock, Ursula von Rüden, Christina Merkel, Boris Orth, Volker Stollorz, Michael Ramharter, Michael Bosnjak, Saad Omer

**Affiliations:** 1 Institut für Medizinische Informationsverarbeitung, Biometrie und Epidemiologie (IBE), Lehrstuhl für Public Health und Versorgungsforschung, Ludwig-Maximilians-Universität (LMU) München, München, Deutschland; 2 Pettenkofer School of Public Health München, München, Deutschland; 3 Institut für Epidemiologie, Sozialmedizin und Gesundheitssystemforschung, Medizinische Hochschule Hannover, Hannover, Deutschland; 4 Bundeszentrale für gesundheitliche Aufklärung (BZgA), Köln, Deutschland; 5 Referat Q3 – Evaluation, Methoden, Forschungsdaten, Bundeszentrale für gesundheitliche Aufklärung (BZgA), Maarweg 149–165, 50825 Köln, Deutschland

**Keywords:** Psychische Gesundheit, Stress, Schule, Pandemie, Eindämmungsmaßnahmen, Mental health, Stress, School, Pandemic, Containment measures

## Abstract

**Hintergrund:**

Eltern stehen während der COVID-19-Pandemie vor einer Vielzahl persönlicher Herausforderungen, während sie gleichzeitig mit schulbezogenen Maßnahmen zur Pandemieeindämmung konfrontiert werden.

**Zielsetzung:**

Dieser Beitrag fokussiert auf die Belastung von Eltern mit Kindern im Schulalter über verschiedene Phasen der COVID-19-Pandemie in Deutschland und identifiziert besonders vulnerable Subgruppen.

**Methoden:**

Die COSMO-Studie ist eine repetitive Querschnittsstudie zur Erfassung der psychosozialen Lage der Bevölkerung in Deutschland während der Pandemie, mit einer Stichprobengröße von ca. *n* = 1000 Befragten pro Erhebungswelle. COSMO-Daten zur allgemeinen und elternspezifischen Belastung wurden von März 2020 bis Januar 2021 quantitativ analysiert.

**Ergebnisse:**

Während der ersten COVID-19-Welle waren Eltern mit Kindern im Schulalter – verglichen mit der allgemeinen Studienpopulation – signifikant stärker belastet. Die Belastung nahm jedoch von März/April bis Juni 2020 deutlich ab. Während der zweiten COVID-19-Welle im Januar 2021 war die Belastung über alle Gruppen hinweg homogen hoch. Folgende Faktoren waren mit einer höheren Belastung assoziiert: Alleinerziehendenstatus, niedriges Haushaltseinkommen, eine chronische Erkrankung, eine COVID-19-Infektion sowie ein Migrationshintergrund; wobei diese Faktoren nicht über alle Erhebungswellen hinweg signifikant waren. Mütter gaben an, stärker von elternspezifischen Belastungen betroffen zu sein als Väter.

**Schlussfolgerung:**

Schulbasierte Maßnahmen zur Infektionskontrolle müssen sorgfältig gegen die Auswirkungen auf die elterliche Belastung mit nachfolgenden negativen Auswirkungen auf das Familiensystem abgewogen werden.

**Zusatzmaterial online:**

Zusätzliche Informationen sind in der Online-Version dieses Artikels (10.1007/s00103-021-03453-3) enthalten.

## Abstract (en)

**Background:**

Parents face a variety of personal challenges during the COVID-19 pandemic, while simultaneously being confronted with additional, school-related pandemic containment measures.

**Objectives:**

To investigate burden in parents of school-aged children across different phases of the COVID-19 pandemic in Germany and to identify particularly affected subgroups.

**Methods:**

The COSMO project is a repetitive cross-sectional survey monitoring the psychosocial situation of the population in Germany during the pandemic with a sample size of approximately *n* = 1000 respondents per survey wave. A quantitative analysis of COSMO data was conducted using closed survey questions on the item “burden” as the main outcome, and, if applicable, on parenthood-associated burden from March 2020 until January 2021.

**Results:**

During the first COVID-19 wave, parents of school-aged children were significantly more burdened compared to the general study population. However, burden decreased significantly from March/April to June 2020. During the second COVID-19 wave in January 2021, burden was homogeneously high across all groups. Single parenthood, a low household income, having a chronic health condition, a COVID-19 infection and a migration background were associated with higher burden, although none of these factors was consistently significant across the survey waves. Mothers reported to be more affected by parenthood-related burden than fathers.

**Conclusions:**

School measures for infection control have to be weighed carefully against the psychological impact on parental burden with subsequent negative impact on the family system.

An English full-text version of this article is available at SpringerLink as Supplementary Information.

## Einleitung

Der Anstieg der COVID-19-Infektionen ab März 2020 führte in Deutschland, wie auch international, zu umfangreichen nichtpharmazeutischen Eindämmungsmaßnahmen. Dazu gehörten u. a. Maskentragen, wiederholte Kontaktbeschränkungen, Test- und Quarantäneverordnungen sowie die Schließung großer Teile des öffentlichen Lebens. Im Vergleich zu anderen Bevölkerungsgruppen sahen sich Kinder und ihre Bezugspersonen mit zusätzlichen, nämlich schulbezogenen Maßnahmen zur Pandemieeindämmung konfrontiert. Den Daten des UNESCO-Education-Response-Panels zufolge gab es am 01.04.2020 landesweite Schulschließungen in 173 Ländern, von denen 84,3 % aller eingeschriebenen Lernenden über alle Bildungsstufen hinweg und damit auch deren Eltern betroffen waren [1]. Der Schulunterricht wurde entweder durch Distanzunterricht bzw. elternbetreutes Homeschooling ersetzt oder er fiel aus.

In diesen schwierigen Zeiten waren Eltern mit einer Vielzahl von Herausforderungen konfrontiert: dem Erhalt der eigenen Gesundheit und des psychischen Wohlbefindens, der Aufrechterhaltung des Berufslebens unter pandemischen Einschränkungen, der Organisation der Beschulung ihrer Kinder und dem Bedürfnis ihrer Kinder nach emotionaler Unterstützung. Letzteres hat durch die COVID-19-Pandemie im Allgemeinen [2–4], aber vor allem durch die Schulschließungen und den damit einhergehenden Verlust von sozialen Kontakten sowie den Verlust von Bildungs‑, Bewegungs- und Spielmöglichkeiten besonders zugenommen [5].

Aus vergangenen Epidemien ist bekannt, dass eine solche Konstellation Familien einem Risiko für psychische Gesundheitsprobleme aussetzt: Eine Studie, die H1N1, SARS und die Vogelgrippe untersuchte, stellte fest, dass isolierte oder in Quarantäne befindliche Eltern und deren Kinder ein erhöhtes Risiko haben, eine posttraumatische Belastungsstörung (PTBS) zu entwickeln. Die elterliche und die kindliche psychische Gesundheit standen dabei im engen Zusammenhang: Bei Eltern mit erhöhtem Risiko für eine PTBS wiesen 87,5 % der Kinder ebenfalls entsprechende Symptome auf [6].

Diese Verflechtung von elterlicher und kindlicher psychischer Gesundheit beruht auf der Bindung zwischen Eltern und Kindern innerhalb eines Familiensystems, die für eine gesunde allgemeine kindliche Entwicklung wichtig ist [7, 8]. Wenn die Gesundheit der Kinder in der Pandemie thematisiert wird, muss daher auch die Gesundheit von Eltern in den Fokus rücken. Im Vergleich zu Kindern wurde die elterliche Gesundheit allerdings während der COVID-19-Pandemie bisher weniger intensiv erforscht. Erste nationale [9–11] und internationale [12–18] Ergebnisse deuten jedoch darauf hin, dass Eltern von den Pandemiemaßnahmen besonders betroffen sind.

In den ersten Monaten der Pandemie zeigte sich, dass sich mit Änderung der Maßnahmen zur Pandemiebekämpfung auch die Raten von Angst, Depression und Distress der Bevölkerung in Deutschland veränderten [19]. Daten über Veränderungen der Situation von Eltern zu verschiedenen Zeitpunkten mit unterschiedlichen Maßnahmen sind jedoch noch rar.

Ziele dieses Beitrags sind deshalb i) mithilfe der COSMO-Daten die Belastung der Eltern über die verschiedenen Phasen der Pandemie hinweg und in Bezug auf gegebene Kontextfaktoren zu analysieren, ii) in der Elterngruppe besonders belastete Subgruppen zu identifizieren, die möglicherweise eine intensivere Unterstützung benötigen, und iii) zu verstehen, welche Aspekte der veränderten Alltagsbedingungen als besonders belastend wahrgenommen wurden, um diesen in einer nächsten Pandemiephase möglicherweise besser begegnen zu können.

## Methoden

### Studiendesign – das COSMO-Projekt

Das Projekt COVID-19-Snapshot-Monitoring(COSMO) ist eine repetitive Querschnittsstudie mit dem Ziel, die psychosoziale Situation der deutschen Bevölkerung im Verlauf der Pandemie zu erfassen. In wöchentlichen bis zweiwöchentlichen Abständen (sog. Wellen) werden seit März 2020 ca. *n* = 1000 Personen im Alter von 18 bis 74 Jahren zu ihrer individuellen psychosozialen Situation, ihrem Wissen über COVID-19 sowie zu ihren Einstellungen gegenüber verschiedenen Institutionen, Behörden und Maßnahmen zur Eindämmung der Pandemie befragt. Für die Stichprobenauswahl verwendet das Projekt ein nichtprobabilistisches Quotenverfahren und strebt eine repräsentative Verteilung der Teilnehmenden in Bezug auf Alter und Geschlecht (gekreuzt) sowie Bundesland an. Die Teilnehmenden werden über das Online-Access-Panel des Markt- und Sozialforschungsunternehmens ‚respondi‘ rekrutiert und erhalten eine Vergütung für ihre Teilnahme [20].

COSMO ist ein Gemeinschaftsprojekt der Universität Erfurt, des Robert Koch-Instituts, der Bundeszentrale für gesundheitliche Aufklärung, des Leibniz-Instituts für Psychologie, des Science Media Centers, des Bernhard-Nocht-Instituts für Tropenmedizin und des US-amerikanischen Yale Institute for Global Health [21]. Während einige Items fortlaufend abgefragt werden, ändern sich die meisten Variablen im Laufe des Projekts und werden daher nur in unregelmäßigen Abständen erhoben, um sich den Veränderungen von Prioritäten im Verlauf der Pandemie anzupassen. Weitere Details sind im Studienprotokoll beschrieben [22], wobei sich das Projekt seit dessen Veröffentlichung bereits weiterentwickelt hat.

### Auswertungszeitpunkte

**Figure 1:**
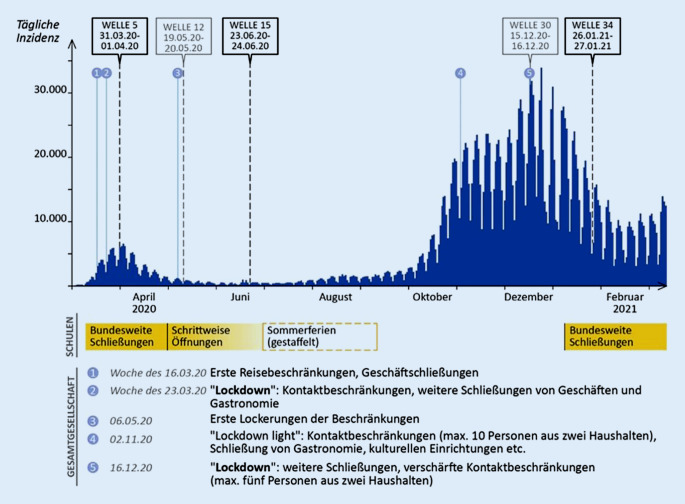

Das Pandemiegeschehen in Deutschland verlief bis zum heutigen Zeitpunkt in verschiedenen, durch sogenannte Wellen charakterisierten Phasen ab. Die erste COVID-19-Welle wurde von Anfang März 2020 bis Mitte Mai 2020 beobachtet. Die zweite COVID-19-Welle folgte Ende September und dauerte bis Ende Februar 2021 [23]. Um die subjektiv empfundene Belastung im Verlauf der Pandemie zu analysieren, wurden die COSMO-Erhebungswellen 5 (31.03./01.04.2020), 15 (23./24.06.2020) und 34 (26./27.01.2021) herangezogen (Abb. 1).

Die gewählten Daten repräsentieren Zeitpunkte, an denen in allen Bundesländern in Deutschland ähnliche Regelungen zum Schulbetrieb galten: COSMO-Welle 5 fand während des ersten Lockdowns ab Mitte März 2020 statt, als Schulen in allen Bundesländern geschlossen waren [24]. Während COSMO-Welle 15 waren alle Schulen zum Präsenzunterricht unter verschiedenen Eindämmungsmaßnahmen zurückgekehrt, wobei sich ein Bundesland (Mecklenburg-Vorpommern) bereits in den Sommerferien befand [25, 26]. Die Schulen wurden in allen Bundesländern in der Woche vom 14.12.2020 abermals zugunsten von Fernunterricht geschlossen [27]. COSMO-Welle 34 erfasst diese Situation. Während der Schulschließungen existierte zu jedem Zeitpunkt eine Notbetreuung für Kinder, deren Eltern in systemrelevanten Berufen arbeiteten.

Zusätzlich wurde eine Reihe von Fragen zur elternspezifischen Belastung in den COSMO-Wellen 12 (19./20.05.2020) und 30 (15./16.12.2020) ausgewertet, die nur zu jenen beiden Zeitpunkten erfasst wurden. COSMO-Welle 12 stellt die Situation während der schrittweisen Wiederöffnungsphase nach der ersten COVID-19-Welle dar. Welle 30 wurde unmittelbar nach Ankündigung der bundesweiten Schulschließungen im Dezember 2020 erhoben.

### Variablenerhebung

#### Zielvariable

Als Hauptuntersuchungsgröße dient das „allgemeine Belastungsempfinden“. Dabei wurden die Studienteilnehmenden gefragt, ob sie ihre persönliche Situation momentan als belastend empfinden (Ja/Nein, binär).

#### Einschlusskriterien und demografische Kovariablen

In zeitlicher Anlehnung an die aktuelle S3-Leitlinie zur Prävention und Kontrolle der SARS-CoV-2-Übertragung in Schulen fokussieren wir hier auf Effekte für Eltern/Betreuungspersonen durch Schulmaßnahmen bzw. -schließungen [28]. Daher betrachten wir die Belastung von Eltern minderjähriger, potenziell schulpflichtiger Kinder im Alter von 6–17 Jahren neben deskriptiven Daten von Eltern jüngerer Kinder (0–5 Jahre) oder volljähriger Kinder bzw. kinderlosen Teilnehmenden.

Berücksichtigt in den Analysen wurden Geschlecht und Alter von teilnehmenden Eltern sowie Alter der jeweiligen Kinder. Zusätzlich wurden die Eltern gefragt, ob sie alleinerziehend sind.

#### Sozioökonomische Kovariablen

Der Bildungshintergrund wurde anhand der Dauer der Schulbildung erfasst. Als weitere Kovariablen wurden die Haushaltsgröße und ein Migrationshintergrund berücksichtigt. Ab Welle 15 wurden zudem der Erwerbsstatus und das Haushaltsnettoeinkommen der Teilnehmenden erhoben.

#### Gesundheitsbezogene Kovariablen

Die Teilnehmenden wurden gefragt, ob sie eine chronische Erkrankung haben, ob sie sich der Risikogruppe für COVID-19-Infektionen zuordnen (ab Welle 15) und ob sie eine COVID-19-Infektion hatten oder haben.^1^

#### Variablen zur Erhebung der elternspezifischen Belastung

Um besonders belastende Aspekte der Elternschaft während der COVID-19-Pandemie zu charakterisieren, wurden 8 Items abgefragt, die elternspezifische Herausforderungen während der Pandemie messen sollten. Dieses Itemset wurde nur in Welle 12 (Mai 2020) und 30 (Dezember 2020) erhoben. Es versucht außerdem die Intensität der elternspezifischen Belastungen durch die Aussagen: a) „Ich fühle mich als Elternteil in der aktuellen Situation überfordert“ und b) „Die aktuelle Situation bringt unsere Familie an den Rand ihrer Kräfte^2^“, abzubilden.

#### Statistische Analysen

Die Gruppenaufteilung erfolgte nach i) Eltern mit Kindern im Schulalter als Hauptinteressengruppe, ii) Eltern mit jüngeren Kindern und iii) Erwachsene ohne minderjährige Kinder oder ohne Kinder im Allgemeinen. Um die Entwicklung der Belastung bei Eltern mit Kindern im Schulalter über die 3 COSMO-Wellen 5, 15 und 34 im Vergleich zu den anderen Gruppen zu analysieren, wurden die absoluten und relativen Häufigkeiten der subjektiv belasteten Befragten für jede Gruppe und Welle berechnet. Um die Verteilung der Belastung pro Welle im Querschnitt zu analysieren, wurde Cramers V verwendet und es wurden die *p*-Werte angegeben. Für den Vergleich im Zeitverlauf wurden Odds Ratios (ORs) und entsprechende 95 %-Konfidenzintervalle (95 % KI) berechnet. Um zu prüfen, ob die gewählten Erhebungszeitpunkte repräsentativ für die jeweilige Phase der Pandemie sind, wurde zudem eine Sensitivitätsanalyse durchgeführt, in der die subjektiv empfundene Belastung von Eltern mit der der allgemeinen Studienpopulation zu allen verfügbaren Zeitpunkten verglichen wurde.

Zur Identifikation besonders betroffener Subgruppen innerhalb der Hauptinteressengruppe wurden deskriptive Statistiken berechnet und eine univariate logistische Regression durchgeführt. Basierend auf vorausgegangen Publikationen wurden für eine multivariate logistische Regression die Variablen Alter, Geschlecht, Bildungshintergrund und Migrationshintergrund der Eltern in das Modell aufgenommen [4]. Wir berichten ORs, 95 % KI und Nagelkerkes R. Die Anpassungsgüte wurde mit dem Hosmer-Lemeshow-Test kontrolliert.

Um in einer Zusatzanalyse die elternspezifische Belastung bei Eltern von Kindern im Schulalter zu untersuchen, wurden deskriptive Statistiken für die COSMO-Erhebungswellen 12 und 30 berechnet und die Belastung von Müttern und Vätern mithilfe von Mann-Whitney-U-Tests verglichen. Als relevantes Maß für die Effektstärke wurde der Korrelationskoeffizient r herangezogen.

Bei allen Berechnungen wurde ein *p*-Wert <0,05 als signifikant erachtet. Aufgrund des explorativen Charakters dieser Studie verzichteten wir auf eine Anpassung des *p*-Werts für Mehrfachtestung. Alle Analysen wurden mit IBMs SPSS Version 27.0 (IBM, Armonk, NY, USA) durchgeführt.

## Ergebnisse

### Stichprobenmerkmale

Tab. 1 zeigt die Stichprobenmerkmale der gesamten Studienpopulation sowie von Eltern mit Kindern im Schulalter. Die Merkmale der Gruppe der Eltern mit jüngeren Kindern und der Erwachsenen ohne minderjährige Kinder bzw. ohne Kinder sind als Onlinematerial verfügbar (Tab. A1).

**Table 1:** 

Stichprobenbeschreibung	Welle 5 (31.03./01.04.2020)				Welle 15 (23./24.06.2020)				Welle 34 (26./27.01.2021)			
	Eltern mit Kindern 6–17 J.		Gesamt		Eltern mit Kindern 6–17 J.		Gesamt		Eltern mit Kindern 6–17 J.		Gesamt	
	*n*/MW	%/SD	*n*/MW	%/SD	*n*/MW	%/SD	*n*/MW	%/SD	*n*/MW	%/SD	*n*/MW	%/SD
Total	188	100,0	1028	100,0	184	100,0	993	100,0	171	100,0	1001	100,0
*Geschlecht*												
Männlich	100	53,2	507	49,3	89	48,4	483	48,6	85	49,7	504	50,3
Weiblich	88	46,8	521	50,7	95	51,6	510	51,4	86	50,3	497	49,7
*Alter der Teilnehmenden (in Jahren)*	43,26	8,0	45,86	16,0	41,49	8,4	45,81	15,5	41,31	9,5	44,5	15,5
*Alter der Teilnehmenden (kategorial)*												
18–29 J.	9	4,8	199	19,4	11	6,0	178	17,9	14	8,2	192	19,2
30–39 J.	47	25,0	160	15,6	65	35,3	221	22,3	60	35,1	243	24,3
40–49 J.	92	48,9	234	22,8	77	41,8	166	16,7	63	36,8	150	15,0
50+ J.	40	21,3	435	42,3	31	16,8	428	43,1	34	19,9	416	41,6
*Alter der Kinder (Mehrfachnennungen möglich)*												
6–9 J.	81	43,1	n.a.	n.a.	84	45,7	n.a.	n.a.	75	43,9	n.a.	n.a.
10–13 J.	69	36,7	n.a.	n.a.	74	40,2	n.a.	n.a.	74	43,3	n.a.	n.a.
14–17 J.	84	44,7	n.a.	n.a.	74	40,2	n.a.	n.a.	63	36,8	n.a.	n.a.
*Alleinerziehend*												
Ja	33	17,6	n.a.	n.a.	36	19,6	n.a.	n.a.	29	17,0	n.a.	n.a.
Nein	155	82,4	n.a.	n.a.	148	80,4	n.a.	n.a.	142	83,0	n.a.	n.a.
*Dauer der Schulbildung*												
Bis zu 9 J.	14	7,4	104	10,1	13	7,1	112	11,3	14	8,2	119	11,9
Mind. 10 J., ohne Abitur	52	27,7	360	35,0	67	36,4	340	34,2	55	32,2	309	30,9
Mind. 10 J., mit Abitur	122	64,9	564	54,9	104	56,5	541	54,5	102	59,6	573	57,2
*Erwerbstätig*												
Ja	n. e.	n. e.	n. e.	n. e.	150	81,5	659	66,4	149	87,1	692	69,1
Nein	n. e.	n. e.	n. e.	n. e.	34	18,5	334	33,6	22	12,9	309	30,9
*Nettohaushaltseinkommen*												
<1250 €	n. e.	n. e.	n. e.	n. e.	8	4,3	142	14,3	8	4,7	115	11,5
1250–2249 €	n. e.	n. e.	n. e.	n. e.	32	17,4	249	25,1	27	15,8	245	24,5
2250–3999 €	n. e.	n. e.	n. e.	n. e.	88	47,8	352	35,4	83	48,5	367	36,7
4000+ €	n. e.	n. e.	n. e.	n. e.	45	24,5	170	17,1	46	26,9	199	19,9
Keine Angabe	n. e.	n. e.	n. e.	n. e.	11	6,0	80	8,1	7	4,1	75	7,5
*Migrationshintergrund*												
Ja	32	17,0	149	14,5	31	16,8	150	15,1	26	15,2	187	18,7
Nein	155	82,4	876	85,2	151	82,1	839	84,5	143	83,6	811	81,0
Weiß nicht	1	0,5	3	0,3	2	1,1	4	0,4	2	1,2	3	0,3
*Haushaltsgröße*												
Nur ich	13	6,9	269	26,2	4	2,2	262	26,4	10	5,8	231	23,1
2 Personen	23	12,2	400	38,9	19	10,3	387	39,0	12	7,0	404	40,4
3–4 Personen	127	67,6	313	30,4	128	69,6	293	29,5	114	66,7	302	30,2
5 oder mehr Pers.	25	13,3	46	4,5	33	17,9	51	5,1	35	20,5	61	6,1
Keine Angabe	0	0	0	0	0	0	0	0	0	0	3	0,3
*Chronische Erkrankung*												
Ja	55	29,3	336	32,7	57	31,0	338	34,0	50	29,2	332	33,2
Nein	127	67,6	651	63,3	122	66,3	632	63,6	116	67,8	634	63,3
Weiß nicht	6	3,2	41	4,0	5	2,7	23	2,3	5	2,9	35	3,5
*Zugehörigkeit zur Risikogruppe für COVID-19*												
Ja	n. e.	n. e.	n. e.	n. e.	76	41,3	518	52,2	42	24,6	349	34,9
Nein	n. e.	n. e.	n. e.	n. e.	108	58,7	475	47,8	113	66,1	572	57,1
Weiß nicht	n. e.	n. e.	n. e.	n. e.	0	0	0	0	16	9,4	80	8,0
*Eigene COVID-19-Infektion (5 und 15/34)*^*a*^												
Ja, bestätigt/Ja	6	3,2	9	0,9	1	0,5	6	0,6	13	7,6	56	5,6
Ja, noch nicht bestätigt/Nein	3	1,6	11	1,1	4	2,2	11	1,1	158	92,4	945	94,4
Nein/–	159	84,6	868	84,4	154	83,7	882	88,8	–	–	–	–
Ja, genesen/–	n. e.	n. e.	n. e.	n. e.	7	3,8	10	1,0	–	–	–	–
Weiß nicht/–	20	10,6	140	13,6	18	9,8	84	8,5	–	–	–	–

*MW* Mittelwert, *SD* Standardabweichung, *n.a.* nicht anwendbar, *n.* *e.* nicht erhoben

^a^Hier hat sich der Modus der Datenerhebung im Laufe des Projekts geändert (COSMO-Wellen 5 und 15: „Ja, Diagnose bestätigt“, „Ja, Diagnose noch nicht bestätigt“, „Ja, überstanden“, „Nein“ und „Weiß nicht“, Welle 34: „Ja“, „Nein“)

Welle 5 umfasste 1028 Teilnehmende, darunter 188 Eltern von Kindern im Schulalter (18,3 % der gesamten Studienpopulation). Die Wellen 15 und 34, mit insgesamt 993 und 1001 Teilnehmenden, hatten ähnliche Anteile von Eltern schulaltriger Kinder, 184 Eltern in Welle 15 (18,5 %) und 171 Eltern in Welle 34 (17,1 %). Eltern zählten sich seltener zur Risikogruppe für COVID-19, lebten im Durchschnitt in größeren Haushalten und verfügten über ein höheres Haushaltsnettoeinkommen.

### Elterliche Belastung in verschiedenen Phasen der Pandemie

**Figure 2:**
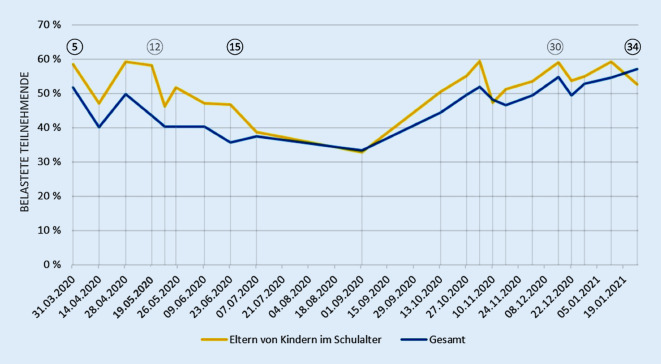

Abb. 2 zeigt die Entwicklung der subjektiv empfundenen Belastung bei Eltern mit schulaltrigen Kindern über alle verfügbaren Erhebungszeitpunkte in COSMO von Welle 5 bis 34. Mit den Daten der Welle 5 wird gut das anfängliche Niveau der Belastung erfasst. In Welle 15 wird der abnehmende Trend deutlich. Die niedrigsten Belastungswerte sind allerdings im Juli und September 2020, zum Zeitpunkt der Sommerferien, zu beobachten. Hier waren auch die Belastungen der Eltern mit Kindern im Schulalter und der gesamten Studienpopulation fast identisch. Welle 34 zeigt die gestiegenen Belastungsniveaus während der zweiten COVID-19-Welle, bildet aber insofern eine Ausnahme, als dass die elterlichen Belastungsniveaus während der zweiten COVID-19-Welle sonst meist etwas über denen der Allgemeinbevölkerung lagen. Ausschläge nach unten in der Woche nach Ostern und um Weihnachten 2020 deuten auf eine Entlastung durch die Feiertage für die gesamte Studienpopulation hin.

**Figure 3:**
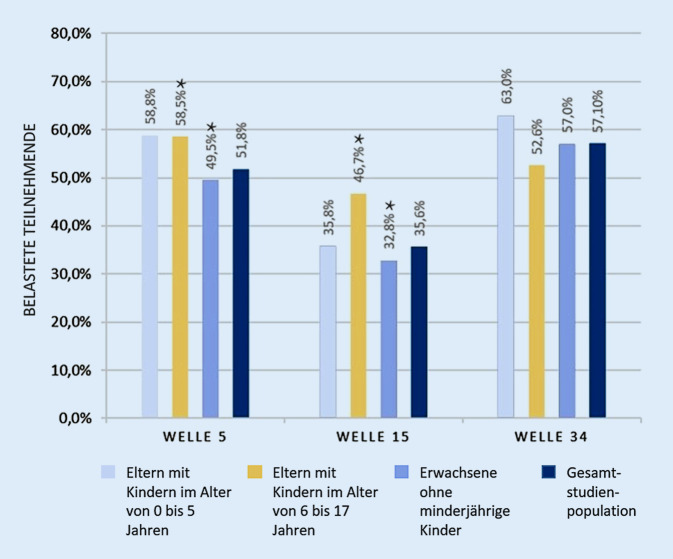

Abb. 3 gibt den Anteil der Studienteilnehmenden in den COSMO-Wellen 5, 15 und 34 wieder, die ihre aktuelle Situation als belastend empfinden, stratifiziert nach Subgruppen.

Während der zweiten COVID-19-Welle stieg der Anteil der belasteten Personen der gesamten Studienpopulation an und lag auf einem höheren Niveau im Vergleich zur ersten COVID-19-Welle (51,8 % vs. 35,6 % vs. 57,1 %). Dabei waren alle beobachteten Veränderungen statistisch signifikant. Ein ähnlicher Verlauf kann auch bei Eltern von schulaltrigen Kindern beobachtet werden, mit einem signifikanten Rückgang von März zu Juni 2020 (Welle 5 zu 15). Der Anstieg von Welle 15 bis Welle 34 war allerdings nicht signifikant.

Im Querschnittsvergleich fühlten sich Eltern mit Kindern im Schulalter in Welle 5 und 15 signifikant stärker belastet als die Gesamtbevölkerung (58,5 % bzw. 46,7 % gegenüber 51,8 % und 35,6 %). In Welle 34 bilden Eltern mit Kindern im Schulalter die am wenigsten belastete Gruppe (52,6 % im Vergleich zu 57,1 %). Allerdings war Cramers V in dieser Welle im Januar 2021 für keine Gruppe signifikant. Weitere detaillierte Ergebnistabellen finden sich im Onlinematerial (Tab. A2 und A3).

### Elterliche Belastung in verschiedenen Subgruppen

Tab. 2 stellt den Anteil der subjektiv belasteten Eltern mit Kindern im Schulalter in verschiedenen Subgruppen dar sowie die Ergebnisse der univariaten logistischen Regressionsanalyse.

**Table 2:** 

	Welle 5 (31.03./01.04.2020)			Welle 15 (23./24.06.2020)			Welle 34 (26./27.01.2021)		
	Belastung = „Ja“ (%)	OR	95 % KI	Belastung = „Ja“ (%)	OR	95 % KI	Belastung = „Ja“ (%)	OR	95 % KI
*Geschlecht*									
Männlich (Referenz)	56,0	–	–	49,4	–	–	45,9	–	–
Weiblich	61,4	1,25	(0,70–2,24)	44,2	0,81	(0,45–1,45)	59,3	1,72	(0,94–3,15)
*Alter der Teilnehmenden*									
18–29 J.	44,4	0,59	(0,14–2,54)	54,5	1,13	(0,28–4,47)	28,6	0,40	(0,11–1,53)
30–39 J.	66,0	1,43	(0,60–3,42)	36,9	0,55	(0,23–1,31)	56,7	1,31	(0,56–3,04)
40–49 J.	56,5	0,96	(0,45–2,04)	51,9	1,10	(0,44–2,33)	55,6	1,25	(0,54–2,88)
50 J.+ (Referenz)	57,5	–	–	51,6	–	–	50,0	–	–
*Alter der Kinder (Mehrfachnennungen möglich)*^*a*^									
6–9 J.	61,7	1,26	(0,70–2,28)	48,8	1,17	(0,65–2,09)	53,3	1,05	(0,57–1,93)
10–13 J.	62,3	1,28	(0,70–2,36)	40,5	0,66	(0,36–1,19)	48,6	0,75	(0,41–1,38)
14–17 J.	52,4	0,63	(0,35–1,14)	54,1	1,64	(0,90–2,96)	49,2	0,81	(0,43–1,50)
*Alleinerziehend*									
Ja	66,7	1,52	(0,69–3,36)	63,9	**2,39**	**(1,12–5,07)**	69,0	2,29	(0,97–5,36)
Nein (Referenz)	56,8	–	–	42,6	–	–	49,3	–	–
*Dauer der Schulbildung*									
Bis zu 9 J.	57,1	0,99	(0,32–3,03)	38,5	0,76	(0,23–2,47)	50,0	0,86	(0,28–2,61)
Mind. 10 J., ohne Abitur	61,5	1,19	(0,61–2,31)	50,7	1,25	(0,68–2,31)	50,9	0,89	(0,46–1,71)
Mind. 10 J., mit Abitur (Referenz)	57,4	–	–	45,2	–	–	53,9	–	–
*Erwerbstätig*									
Ja (Referenz)	n. e.	n. e.	n. e.	45,3	–	–	54,4	–	–
Nein	n. e.	n. e.	n. e.	52,9	1,36	(0,64–2,86)	40,9	0,58	(0,23–1,44)
*Nettohaushaltseinkommen*									
<1250 €	n. e.	n. e.	n. e.	87,5	**12,69**	**(1,43–112,51)**	25,0	0,43	(0,08–2,38)
1250–2249 €	n. e.	n. e.	n. e.	53,1	2,05	(0,82–5,18)	51,9	1,40	(0,54–3,63)
2250–3999 €	n. e.	n. e.	n. e.	46,6	1,58	(0,75–3,32)	57,8	1,78	(0,86–3,69)
4000+ € (Referenz)	n. e.	n. e.	n. e.	35,6	–	–	43,5	–	–
*Migrationshintergrund*									
Bekannt	68,8	1,70	(0,76–3,83)	61,3	2,03	(0,92–4,48)	69,2	2,28	(0,93–5,58)
Nicht bekannt (Referenz)	56,8	–	–	43,8	–	–	49,7	–	–
*Haushaltsgröße*									
Nur ich (Referenz)	53,8	–	–	50,0	–	–	40,0	–	–
2 Personen	60,9	1,33	(0,34–5,27)	63,2	1,71	(0,20–15,02)	41,7	1,07	(0,19–5,91)
3–4 Personen	56,7	1,12	(0,36–3,53)	43,8	0,78	(0,11–5,70)	57,0	1,99	(0,53–7,44)
5 oder mehr Personen	68,0	1,82	(0,46–7,22)	48,5	0,94	(0,12–7,50)	45,7	1,26	(0,30–5,28)
*Chronische Erkrankung (des Elternteils)*									
Bekannt	65,5	1,51	(0,79–2,90)	63,2	**2,64**	**(1,39–5,03)**	58,0	1,36	(0,70–2,64)
Nicht bekannt (Referenz)	56,7	–	–	39,4	–	–	50,4	–	–
*Zugehörigkeit zur Risikogruppe für COVID-19*									
Bekannt	n. e.	n. e.	n. e.	55,3	1,80	(0,99–3,25)	52,4	0,99	(0,49–1,98)
Nicht bekannt (Referenz)	n. e.	n. e.	n. e.	40,7	–	–	52,7	–	–
*Eigene COVID-19-Infektion (5 und 15/34)*^*b*^									
Ja, gesichert/Ja	66,7	X	X	100,0	X	X	84,6	**5,50**	**(1,18–25,62)**
Ja, noch nicht gesichert/Nein	100,0	X	X	75,0	X	X	50,0	***–***	***–***
Nein/–	57,2	X	X	44,8	X	X	n. e.	n. e.	n. e.
Ja, genesen/–	–	X	X	42,9	X	X	n. e.	n. e.	n. e.
Weiß nicht/–	60,0	X	X	55,6	X	X	n. e.	n. e.	n. e.

Signifikante Ergebnisse sind fett gedruckt

*n.* *e.* nicht erhoben, *X* Aufgrund sehr kleiner Subgruppen wurde auf eine Regressionsanalyse verzichtet

^a^Da bei dieser Variablen mehrere Antwortmöglichkeiten gleichzeitig angegeben werden konnten (Mehrfachnennung möglich), wurde hier zur Berechnung der OR nicht eine Referenzkategorie festgelegt, sondern jeweils die Gruppe, die diese Antwortmöglichkeit gewählt hat, mit der Gruppe verglichen, die diese Antwortoption nicht ausgewählt hat

^b^Hier hat sich der Modus der Datenerhebung im Laufe des Projekts geändert (COSMO Wellen 5 und 15: „Ja, Diagnose bestätigt“, „Ja, Diagnose noch nicht bestätigt“, „Ja, überstanden“, „Nein“ und „Weiß nicht“, Welle 34: „Ja“, „Nein“)

Mehr Mütter als Väter fühlen sich sowohl während der ersten COVID-19-Welle Ende März/Anfang April 2020 (56,0 % vs. 61,4 %) belastet als auch während der zweiten Welle im Januar 2021 (45,9 % vs. 59,3 %). Im Juni 2020 hingegen waren eher Väter als Mütter (49,4 % vs. 44,2 %) belastet, wobei diese Ergebnisse jedoch nicht signifikant waren. Es konnte kein Zusammenhang in Bezug auf Alter der Eltern oder ihrer Kinder hinsichtlich eines erhöhten Belastungsempfinden festgestellt werden. In Welle 15 im Juni 2020 war der Status als alleinerziehend mit einem signifikant höheren Belastungsempfinden assoziiert (OR 2,39; 95 % KI 1,12–5,07). In Welle 5 und 34 war dieser Zusammenhang nicht signifikant.

Bezogen auf die sozioökonomischen Variablen war lediglich ein sehr niedriges Haushaltseinkommen unter 1250 € pro Monat und auch ausschließlich in Welle 15 signifikant mit höherem Belastungsempfinden assoziiert (OR 12,69; 95 % KI 1,43–112,51). Zwar schienen Eltern mit einem Migrationshintergrund über alle Wellen hinweg häufiger belastet zu sein, jedoch weisen die statistischen Tests hier keine Signifikanz aus. Bei den gesundheitsbezogenen Kovariablen war das Bestehen einer chronischen Erkrankung in allen Wellen mit einer höheren Belastung assoziiert (65,5 % vs. 56,7 % in Welle 5; 63,2 % vs. 39,4 % in Welle 15 und 58,0 % vs. 50,4 % in Welle 34), wenn auch der Unterschied nur in Welle 15 statistisch signifikant war (OR 2,64; 95 % KI 1,39–5,03). Für die Gruppe der Eltern, die einer Risikogruppe für COVID-19 angehören, ergaben sich keine signifikanten Ergebnisse. Eine stattgehabte COVID-19-Infektion war hingegen, zumindest in Welle 34, mit einem höheren Belastungsempfinden signifikant assoziiert (84,6 % vs. 50 %; OR 5,50; 95 % KI 1,18–25,62).

Die multivariate Regression (Onlinematerial, Tab. A4) zeigte für Welle 15 (Juni 2020) eine signifikante Assoziation zwischen einem Migrationshintergrund und einem höheren Belastungsempfinden (OR 2,34; 95 % KI 1,03–5,34). Alter, Schulbildung und Geschlecht blieben insignifikant. Nagelkerkes Pseudo‑R^2^ war zu allen Zeitpunkten gering.

### Analyse elternspezifischer Belastung

COSMO-Welle 12 (Mai 2020) umfasste *n* = 184 Eltern von Kindern im Schulalter, darunter 90 Väter (48,9 %) und 94 Mütter (51,1 %), Welle 30 (Dezember 2020) *n* = 188 Eltern von Kindern im Schulalter, darunter 95 Väter (50,5 %) und 93 Mütter (49,5 %). Das Onlinematerial Abb. A1 zeigt für beide Wellen Boxplotdiagramme für jedes Item der elternspezifischen Belastungsvariablen. Die Gründe für die Belastung waren in beiden Wellen recht einheitlich. Die Kinder bei Laune zu halten, ohne dass sie Kontakt zu Gleichaltrigen haben (Median 4,5 in Welle 12 und Median 5,0 in Welle 30), den Schulunterricht zu organisieren (Median 5) und die Großeltern nicht mehr sehen zu können (Median 5) wurden als größte Herausforderung empfunden.

**Figure 4:**
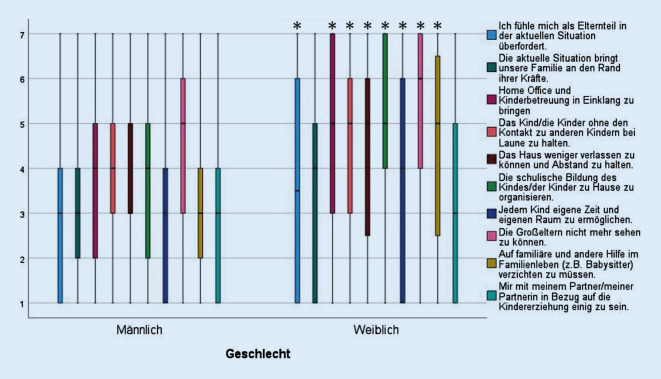

Abb. 4 zeigt für Welle 12 Boxplotdiagramme mit diesem Itemsatz, stratifiziert nach Geschlecht, und offenbart eine meist signifikante, höhere Belastung bei Müttern als bei Vätern. Im Onlinematerial werden die Ergebnisse des Mann-Whitney-U-Tests (Tab. A5) zusammen mit den entsprechenden Ergebnissen für Welle 30 (Tab. A6; Abb. A2) dargestellt, die dieses Bild größtenteils bestätigen.

## Diskussion

In dieser Studie untersuchten wir die Belastung der Eltern zu definierten Zeitpunkten während der COVID-19-Pandemie in Deutschland. Unsere wichtigsten Ergebnisse erbrachten, dass Eltern von Kindern im Schulalter während der ersten COVID-19-Welle und im Anschluss deutlich stärker belastet waren als die allgemeine Studienpopulation. Mütter schienen durch die Elternschaft unter Pandemiebedingungen stärker belastet als Väter.

Unsere Ergebnisse decken sich hierin mit ähnlichen Studien zur Situation von Eltern während der Pandemie in Deutschland. So fanden Rothe et al. im April und Anfang Mai 2020 ein höheres Stressempfinden bei Eltern als bei Erwachsenen, die nicht mit minderjährigen Kindern zusammenleben [10]. Huebener et al. berichteten im Mai und Juni 2020 über einen stärkeren Rückgang der Lebenszufriedenheit bei Eltern von Kindern unter 11 Jahren im Vergleich zu Erwachsenen ohne Kinder [11]. Auch die Analysen von Calvano et al. zeigten, dass Eltern insgesamt gestresster waren als vor der Pandemie [9].

Nach internationalen Studien scheint das Auftreten von erhöhtem elterlichen Stress und Belastung ein weltweites Phänomen während der Pandemie zu sein (z. B. im Vereinigten Königreich [15], in den USA [12–14], in Italien [16, 17] und in Guatemala [18]), wobei ein höheres Stressniveau mit einem jüngeren Alter von Eltern und Kindern assoziiert war.

Als Risikofaktoren für eine höhere Belastung von Eltern konnten wir folgende Aspekte identifizieren: eine chronische Erkrankung, niedriges Haushaltseinkommen, Alleinerziehendenstatus, eine eigene COVID-19-Infektion sowie einen Migrationshintergrund.

Diese Faktoren werden auch in Studien mit ähnlichen Fragestellungen genannt: So zeigten Erhebungen, dass eine psychische Erkrankung (als wichtige Untergruppe chronischer Erkrankungen) mit elterlichem Stress korreliert [10] ebenso wie ein niedriges Haushaltseinkommen [11]. Alleinerziehend zu sein korrelierte auch in internationalen Studien mit elterlichem Stress und Erschöpfung [16, 17]. Die Ergebnisse der COPSY-Studie zeigen, dass ein niedriger elterlicher Bildungsstand und ein elterlicher Migrationshintergrund mit negativen Folgen für die psychische Gesundheit von Kindern während der COVID-19-Pandemie verbunden sind und daher auch bei der Bewertung von Risikofaktoren für die elterliche Belastung relevant sind [4].

In Bezug auf die elternspezifische Belastung durch den Fernunterricht zeigten unsere Analysen, dass die Organisation des Schulunterrichts als eine der größten Herausforderungen wahrgenommen wurde, auch aufgrund von fehlender Unterstützung durch die Großeltern oder Haushaltshilfen. In einer anderen deutschen Studie gaben Eltern die Distanzierungsmaßnahmen, Einschränkungen und geschlossene Kinderbetreuungseinrichtungen als besonders belastend an [9]. Thorell et al. stellten fest, dass das Angebot an Onlineunterricht in vielen Ländern, darunter auch in Deutschland, begrenzt war, sodass die Hauptverantwortung für den Schulunterricht auf die Eltern übertragen wurde [29]. Die Studie zeigte im Frühjahr 2020 bei 57,2 % der deutschen Eltern eine erhöhte elterliche Belastung durch den Heimunterricht, wobei die Belastung bei Eltern von Kindern mit psychischen Erkrankungen noch höher war. Eltern in Deutschland waren somit durch Heimunterricht ähnlich belastet wie Eltern in Italien, Spanien, Belgien und im Vereinigten Königreich. Generell berichteten Eltern in praktisch allen Ländern von einer Zunahme häuslicher Konflikte [29].

Auf der anderen Seite berichtete jedoch eine kleinere Zahl von Eltern auch über positive Veränderungen im Zusammenhang mit der Pandemie, z. B. durch mehr Zeit für die Familie und größere Dankbarkeit [9].

In unserer geschlechterstratifizierten Zusatzanalyse der elternspezifischen Belastung in COSMO für Mai (Welle 12) und Dezember 2020 (Welle 30) konnten wir in beiden Wellen ein ähnliches Belastungsmuster erkennen, wobei insbesondere Mütter stärker belastet waren als Väter. Dies lässt vermuten, dass die psychische Gesundheit von Müttern in besonderem Maße gefährdet ist. So berichten auch mehrere andere Publikationen von einer stärkeren Abnahme der Lebenszufriedenheit bei Müttern im Vergleich zu Vätern [11, 30]. Dies mag nicht nur potenziell ungünstige Auswirkungen auf ihre Gesundheit, sondern auch die soziale und wirtschaftliche Gleichstellung haben [11, 14, 16, 31]. Mütter mit minderjährigen Kindern reduzierten während der COVID-19-Pandemie mehr Stunden bezahlter Arbeit und übernahmen überproportional mehr zusätzliche Betreuungsarbeit. Dies wurde in Deutschland [30, 32, 33] wie auch in anderen Ländern wie den USA [34] und dem Vereinigten Königreich [15, 35, 36] beobachtet. In einer britischen Studie übernahmen Mütter und Väter nur dann den gleichen Anteil an der Kinderbetreuung, wenn die Mütter einer Erwerbsarbeit nachgingen und die Väter gleichzeitig nicht. Außerdem berichteten berufstätige Mütter über einen größeren Anteil an unterbrochener Erwerbstätigkeit als ihre männlichen Kollegen [36]. Russel et al. stellten andererseits fest, dass in den USA Väter stärker gestresst waren als Mütter [12].

Hierbei ist wichtig anzumerken, dass, der Aktualität des Themas geschuldet, viele der genannten Studien unter der Verwendung von Convenience-Sampling-Methoden, also durch willkürliche Stichprobengewinnung, entstanden sind und deren Ergebnisse somit nicht auf repräsentativen Stichproben beruhen, was ihre Aussagekraft einschränken könnte.

### Limitationen

Während die Gesamtstichprobe der COSMO-Studie mit ca. *n* = 1000 Teilnehmenden pro Erhebungswelle eine repräsentative Größe hat, war die für uns relevante Subgruppe, Eltern von schulaltrigen Kindern, jeweils deutlich kleiner, weshalb eine Über- oder Unterschätzung der erkannten Zusammenhänge nicht ausgeschlossen werden kann. Da COSMO darauf abzielt, Momentaufnahmen zu einer breiten Palette von Themen zu erfassen, anstatt eine eingehende Analyse bestimmter Aspekte vorzunehmen, konnten umfangreiche Fragebögen oder standardisierte Diagnoseinstrumente nicht integriert werden. Zudem wurden hier kinderbezogene Daten, mit Ausnahme des Alters, nicht erfasst.

Auch aus der onlinebasierten Datenerhebung von COSMO ergeben sich einige Einschränkungen, die die Aussagekraft unserer Ergebnisse beeinflussen können: So sind Teilnehmende aus niedrigeren sozioökonomischen Gruppen deutlich unter- und Teilnehmende aus höheren sozioökonomischen Gruppen deutlich überrepräsentiert, was die Aussagekraft der Ergebnisse limitiert. Da das Geschlecht hier nur binär erfasst wird, sind andere Geschlechtsidentitäten nicht vertreten. Wenn eine Familie durch ihre derzeitige Situation am Rande ihrer Kräfte ist, ist die Wahrscheinlichkeit, dass ein Elternteil an einer wissenschaftlichen Umfrage teilnimmt, geringer als bei denjenigen, die nicht betroffen sind. Dies könnte dazu geführt haben, dass die Belastungsprävalenz sogar noch unterschätzt wurde.

## Schlussfolgerungen

Diese Studie zeigt, dass besonders Eltern in den verschiedenen Phasen der COVID-19-Pandemie in Deutschland stark belastet waren und sind. Es ist daher wichtig, dass die pandemiebedingten elterlichen Belastungen bei politischen Entscheidungen über Maßnahmen zur Pandemieeindämmung berücksichtigt werden. Darüber hinaus sollten Eltern bei der Ausgestaltung von Angeboten zur psychologischen und praktischen Unterstützung besonders berücksichtigt werden. So wird seitens der Weltgesundheitsorganisation (WHO) für die europäische Region die Entwicklung zielgruppengerechter, bedarfsorientierter Aufklärungs- und Unterstützungsangebote vorgeschlagen [37], die sich insbesondere auch an Eltern aus risikobelasteten sozialen Verhältnissen richten sollten. In Anbetracht der ungleichen Belastung von Müttern und Vätern durch die Pandemie ist es wichtig, dass auch Mütter in politischen Entscheidungsgremien repräsentiert sind und darüber hinaus maßgeschneiderte Unterstützung erhalten.

## Supplementary Information






## Footnotes

1 Hier hat sich der Modus der Datenerhebung im Laufe des Projekts geändert (COSMO Wellen 5 und 15: „Ja, Diagnose bestätigt“, „Ja, Diagnose noch nicht bestätigt“, „Ja, überstanden“, „Nein“ und „Weiß nicht“, Welle 34: „Ja“, „Nein“).

2 „Familie“ bezog sich auf die Personen, mit denen die Befragten ihren Haushalt teilen.
