# Supplementary material for: Burden in parents of school-aged children during different phases of the COVID-19 pandemic in Germany: an analysis within the COVID-19 snapshot monitoring (COSMO) study
Source: Bundesgesundheitsblatt Gesundheitsforschung Gesundheitsschutz. 2021 Nov 26;64(12):1500–11. [Article in German] doi: 10.1007/s00103-021-03453-3 (PMC8617549; doi:10.1007/s00103-021-03453-3)
Supplement: Supplementary file 1 [file 103_2021_3453_MOESM1_ESM.pdf]

Bundesgesundheitsblatt 12/2021

Onlinematerial zum Beitrag:

Belastung von Eltern mit Kindern im Schulalter während verschiedener Phasen der COVID-19-Pandemie in Deutschland: Eine Analyse der COVID-19 Snapshot Monitoring (COSMO)-Daten

Julia Elisabeth Rabe[1,2,3], Hannah Schillok[1,2], Christina Merkel[4], Stephan Voss[1,2], Michaela Coenen[1,2], Freia De Bock[4], Ursula von Rüden[4], Anke Bramesfeld[3], Caroline Jung-Sievers[1,2], COSMO-Gruppe[*]

[1] Institut für Medizinische Informationsverarbeitung, Biometrie und Epidemiologie (IBE), Lehrstuhl für Public Health und Versorgungsforschung, Ludwig-Maximilians-Universität (LMU) München, München, Deutschland

[2] Pettenkofer School of Public Health München, München, Deutschland

[3] Institut für Epidemiologie, Sozialmedizin und Gesundheitssystemforschung, Medizinische Hochschule Hannover, Hannover, Deutschland

[4] Bundeszentrale für gesundheitliche Aufklärung (BZgA), Köln, Deutschland

*Die Mitglieder der COSMO-Gruppe sind in der Danksagung aufgeführt.

Korrespondenzadresse:
Dr. Ursula von Rüden
Referat Q3 - Evaluation, Methoden, Forschungsdaten
Bundeszentrale für gesundheitliche Aufklärung (BZgA)
Maarweg 149-165
50825 Köln
Deutschland
ursula.von-rueden@bzga.de

Inhalte:

Abbildung A1: Elternspezifische Belastung in Welle 12 und 30

Abbildung A2: Vergleich elternspezifischer Belastungen stratifiziert nach Geschlecht in Welle 30

Tabelle A1: Soziodemografische und gesundheitsbezogenen Angaben für Eltern mit Kindern im Alter von 0 bis 5 Jahren sowie für Teilnehmende ohne minderjährige Kinder in den Wellen 5, 15 und 34.

Tabelle A2: Vergleich der Belastung innerhalb der Wellen 5, 15 und 34, inkl. absoluter und relativer Häufigkeiten sowie Cramers V und p-Werten für die näherungsweise Signifikanz.

Tabelle A3: Vergleich der Belastung innerhalb der Hauptgruppen zwischen den Wellen 5, 15 und 34, inkl. Odds Ratios (OR) und 95% Konfidenzintervallen (95% KI).

Tabelle A4: Multivariate logistische Regression für die Belastung in verschiedenen Subgruppen von Eltern mit Kindern im Schulalter mit den Kovariablen Alter, Geschlecht, Schulbildung und Migrationshintergrund.

Tabelle A5: Vergleich elternspezifischer Belastungen zwischen Müttern und Vätern von Kindern im Schulalter in Welle 12 durch den Mann-Whitney-U-Test.

Tabelle A6: Vergleich elternspezifischer Belastungen zwischen Müttern und Vätern von Kindern im Schulalter in Welle 30 durch den Mann-Whitney-U-Test.

Abbildung A1 – Elternspezifische Belastung in Welle 12 und 30
Boxplot-Diagramme für das Variablenset zu elternspezifischer Belastung in Welle 12 (Mai 2020) und Welle 30 (Dezember 2020). Antwortmöglichkeiten für die ersten beiden Items waren 1 - „Trifft überhaupt nicht zu“ bis 7 – „Trifft vollständig zu“, sowie 1 „Gar nicht herausfordernd“ bis 7 – „Äußerst herausfordernd“ für die verbleibenden acht Items.

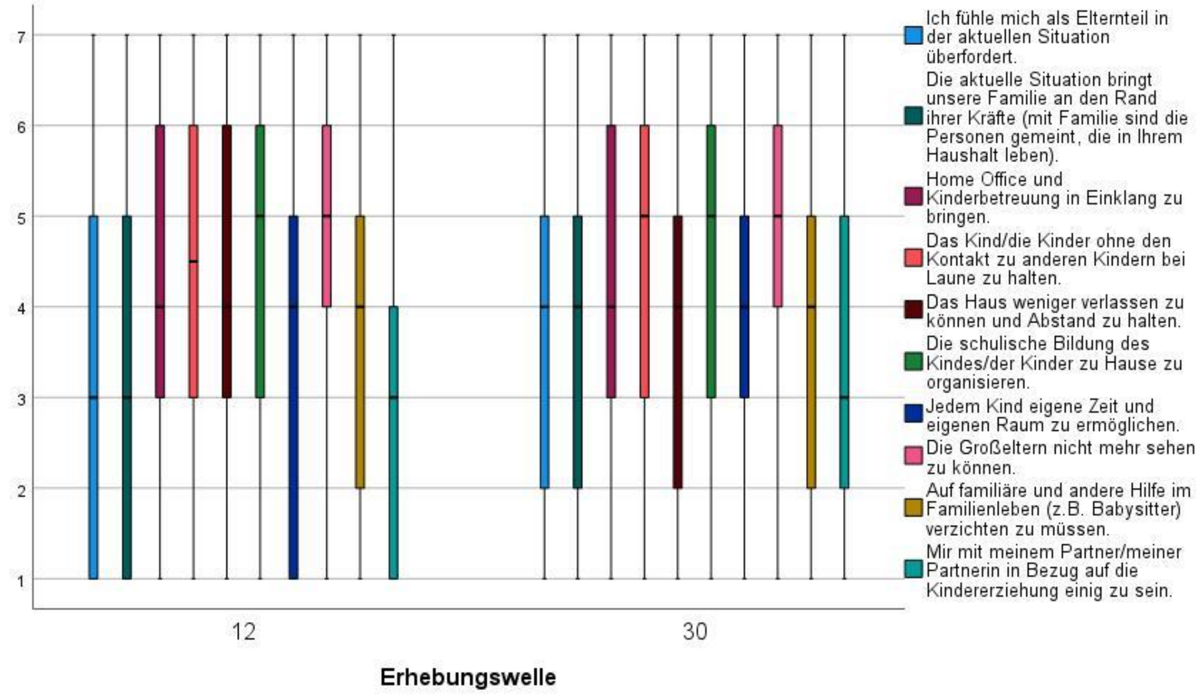

Abbildung A2 – Vergleich elternspezifischer Belastungen stratifiziert nach Geschlecht in Welle 30
Boxplot-Diagramme für das Variablenset zu elterlicher Belastung für Mütter im Vergleich zu Vätern von Kindern im Schulalter in Welle 30 (Dezember 2020). Antwortmöglichkeiten für die ersten beiden Items waren 1 - „Trifft überhaupt nicht zu“ bis 7 – „Trifft vollständig zu“, sowie 1 „Gar nicht herausfordernd“ bis 7 – „Äußerst herausfordernd“ für die verbleibenden acht Items. Signifikante Unterschiede aus den Mann-Whitney-U-Tests sind mit „*“ markiert.

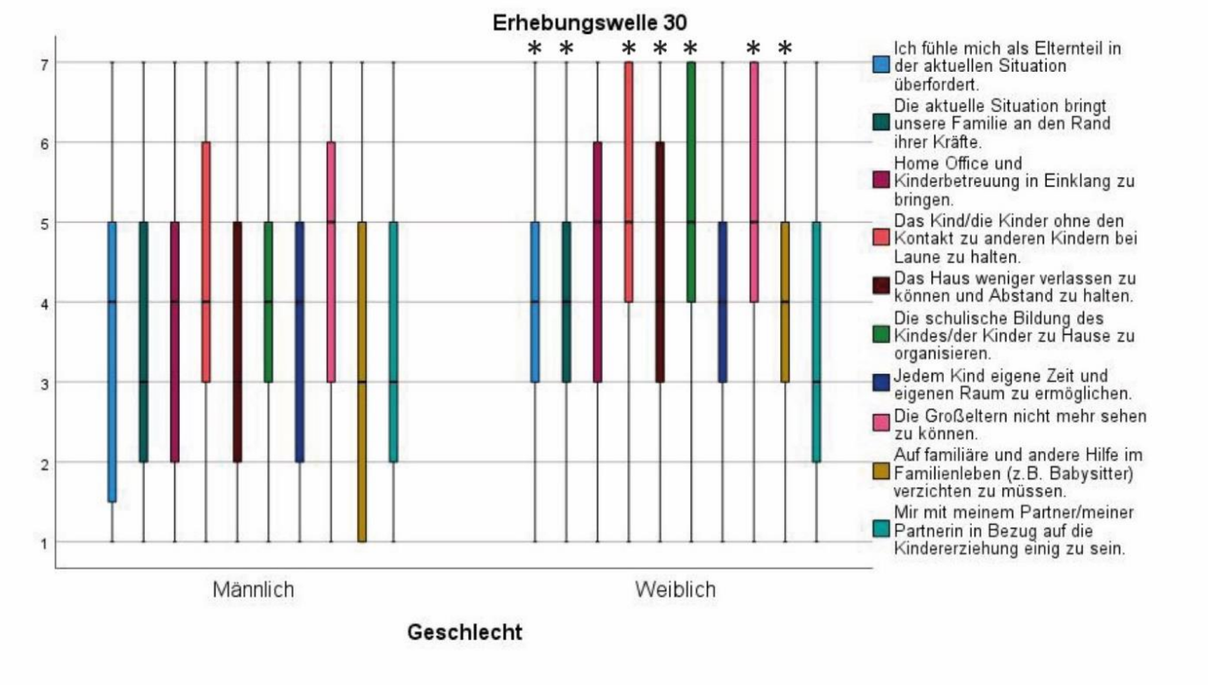

Tabelle A1: Soziodemografische und gesundheitsbezogenen Angaben für Eltern mit Kindern im Alter von 0 bis 5 Jahren sowie für Teilnehmende ohne minderjährige Kinder in den Wellen 5, 15 und 34. MW: Mittelwert. SD: Standardabweichung. n.e.: nicht erhoben.

[a.] Hier hat sich der Modus der Datenerhebung im Laufe des Projekts geändert (COSMO Wellen 5 und 15: "Ja, Diagnose bestätigt", "Ja, Diagnose noch nicht bestätigt", "Ja, überstanden", "Nein" und "Weiß nicht", Welle 34: "Ja", "Nein").

**Stichproben-beschreibung**	Welle 5 (31. März/ 1. April 2020)				Welle 15 (23./24. Juni 2020)				Welle 34 (26./27. Januar 2021)			
	Eltern mit Kindern 0-5 J.		Teilnehmende ohne Kinder <18 J.		Eltern mit Kindern 0-5 J.		Teilnehmende ohne Kinder <18 J.		Eltern mit Kindern 0-5 J.		Teilnehmende ohne Kinder <18 J.	
	n/MW	%/SD	n/MW	%/SD	n/MW	%/SD	n/MW	%/SD	n/MW	%/SD	n/MW	%/SD
Total	131	100,0%	744	100,0%	162	100,0%	698	100,0%	154	100,0%	716	100,0%
**Geschlecht**												
Männlich	60	45,8 %	364	48,9 %	66	40,7 %	350	50,1 %	79	51,3 %	360	50,3 %
Weiblich	71	54,2 %	380	51,1 %	96	59,3 %	348	49,9 %	75	48,7 %	356	49,7 %
**Alter der Teilnehmenden (in Jahren)**	36,28	10,558	47,95	17,293	36,61	10,008	48,52	16,631	36,36	11,392	46,58	16,542
**Alter der Teilnehmenden (kategorial)**												
18-29 J.	31	23,7 %	161	21,6 %	35	21,6 %	136	19,5 %	31	20,1 %	150	20,9 %
30-39 J.	57	43,5 %	71	9,5 %	83	51,2 %	99	14,2 %	90	58,4 %	121	16,9 %
40-49 J.	31	23,7 %	125	16,8 %	31	19,1 %	78	11,2 %	18	11,7 %	76	10,6 %
50+ J.	12	9,2 %	387	52,0 %	13	8,0 %	385	55,2 %	15	9,7 %	369	51,5 %
**Alleinerziehend**												
Ja	16	12,2 %	-	-	20	12,3 %	-	-	15	9,7 %	-	-
Nein	115	87,8 %	-	-	142	87,7 %	-	-	139	90,3 %	-	-
**Dauer der Schulbildung**												
Bis zu 9 J.	11	8,4 %	84	11,3 %	18	11,1 %	84	12,0 %	14	9,1 %	94	13,1 %
Mind. 10 J., ohne Abitur	24	18,3 %	287	38,6 %	41	25,3 %	247	35,4 %	40	26,0 %	225	31,4 %
Mind. 10 J., mit Abitur	96	73,3 %	373	50,1 %	103	63,6 %	367	52,6 %	100	64,9 %	397	55,4 %
**Erwerbstätig**												
Ja	n.e.	n.e.	n.e.	n.e.	126	77,8 %	422	60,5 %	131	85,1 %	448	62,6 %
Nein	n.e.	n.e.	n.e.	n.e.	36	22,2 %	276	39,5 %	23	14,9 %	268	37,4 %
**Nettohaushalts-einkommen**												
<1.250€	n.e.	n.e.	n.e.	n.e.	9	5,6 %	126	18,1 %	5	3,2 %	102	14,2 %
1.250-2.249	n.e.	n.e.	n.e.	n.e.	39	24,1 %	188	26,9 %	34	22,1 %	190	26,5 %
2.250-3.999	n.e.	n.e.	n.e.	n.e.	71	43,8 %	218	31,2 %	73	47,4 %	238	33,2 %
4.000+	n.e.	n.e.	n.e.	n.e.	34	21,0 %	101	14,5 %	31	20,1 %	128	17,9 %
Keine Angabe	n.e.	n.e.	n.e.	n.e.	9	5,6 %	65	9,3 %	11	7,1 %	658	91,9 %
**Migrations-hintergrund**												
Ja	30	22,9 %	94	12,6 %	40	24,7 %	91	13,0 %	36	23,4 %	135	18,9 %
Nein	100	76,3 %	648	87,1 %	121	74,7 %	606	86,8 %	118	76,6 %	580	81,0 %
Weiß nicht	1	0,8 %	2	0,3 %	1	0,6 %	1	0,1 %	-	-	1	0,1 %
**Haushaltsgröße**												
Nur ich	8	6,1 %	249	33,5 %	11	6,8 %	247	35,4 %	12	7,8 %	212	29,6 %
2 Personen	15	11,5 %	363	48,8 %	21	13,0 %	350	50,1 %	14	9,1 %	378	52,8 %
3-4 Personen	88	67,2 %	120	16,1 %	108	66,7 %	85	12,2 %	100	64,9 %	108	15,1 %
5 oder mehr Pers.	20	15,3 %	12	1,6 %	22	13,6 %	16	2,3 %	26	16,9 %	17	2,4 %
Keine Angabe	0	0,0 %	0	0,0 %	0	0,0 %	0	0,0 %	2	1,3 %	1	0,1 %
**Chronische Erkrankung**												
Ja	22	16,8 %	263	35,3 %	38	23,5 %	253	36,2 %	30	19,5 %	259	36,2 %
Nein	100	76,3 %	454	61,0 %	121	74,7 %	428	61,3 %	120	77,9 %	431	60,2 %
Weiß nicht	9	6,9 %	27	3,6 %	3	1,9 %	17	2,4 %	4	2,6 %	26	3,6 %

**Zugehörigkeit zur Risikogruppe für COVID-19**																
Ja	n.e.	n.e.	n.e.	n.e.	40	24,7 %	416	59,6 %	27	17,5 %	289	40,4 %
Nein	n.e.	n.e.	n.e.	n.e.	122	75,3 %	282	40,4 %	117	76,0 %	372	52,0 %
Weiß nicht	n.e.	n.e.	n.e.	n.e.	0	0,0 %	0	0,0 %	10	6,5 %	55	7,7 %
**Eigene COVID-19 Infektion (5&15/34)** [a]												
Ja, bestätigt/ Ja	3	2,3 %	2	0,3 %	2	1,2 %	3	0,4 %	15	9,7 %	32	4,5 %
Ja, noch nicht bestätigt/ Nein	5	3,8 %	4	0,5 %	4	2,5 %	3	0,4 %	139	90,3 %	684	95,5 %
Nein / -	102	77,9 %	634	85,2 %	137	84,6 %	638	91,4 %	-	-	-	-
Ja, genesen / -	n.e.	n.e.	n.e.	n.e.	1	0,6 %	3	0,4 %	-	-	-	-
Weiß nicht / -	21	16,0 %	104	14,0 %	18	11,1 %	51	7,3 %	-	-	-	-

Tabelle A2: Vergleich der Belastung innerhalb der Wellen 5, 15 und 34, inkl. absoluter und relativer Häufigkeiten sowie Cramers V und p-Werten für die näherungsweise Signifikanz. Signifikante Ergebnisse sind fett gedruckt (p<0.05).

Welle 5	Gruppen-größe (N)	Belastung = „Ja“ (n)	Belastung = „Ja“ (%)	Cramers V	Näherungsw. Signifikanz
Eltern von Kindern 0-5 J.	131	77	58,8%	0,05	0,09
Eltern von Kindern 6-17 J.	188	110	58,5%	**0,06**	**0,04**
Keine Kinder <18 J.	744	368	49,5%	**0,08**	**0,01**
Gesamt	131	77	58,8%		

Welle 15	Gruppen-größe (N)	Belastung = „Ja“ (n)	Belastung = „Ja“ (%)	Cramers V	Näherungsw. Signifikanz
Eltern von Kindern 0-5 J.	162	58	35,8%	<0,01	0,97
Eltern von Kindern 6-17 J.	184	86	46,7%	**0,11**	**<0,01**
Keine Kinder <18 J.	698	229	32,8%	**0,09**	**<0,01**
Gesamt	993	354	35,6%		

Welle 34	Gruppen-größe (N)	Belastung = „Ja“ (n)	Belastung = „Ja“ (%)	Cramers V	Näherungsw. Signifikanz
Eltern von Kindern 0-5 J.	154	97	63,0%	0,050	0,11
Eltern von Kindern 6-17 J.	171	90	52,6%	0,041	0,19
Keine Kinder <18 J.	716	408	57,0%	0,005	0,87
Gesamt	1001	572	57,1%		

Tabelle A3: Vergleich der Belastung innerhalb der Hauptgruppen zwischen den Wellen 5, 15 und 34, inkl. Odds Ratios (OR) und 95% Konfidenzintervallen (95% KI). Signifikante Ergebnisse in fett gedruckt ($p<0.05$).

	Welle 15 vs. 5		Welle 34 vs. 5		Welle 34 vs. 15	
	OR	95% KI	OR	95% KI	OR	95% KI
Eltern von Kindern 0-5 J.	**0,39**	**(0,24-0,63)**	1,19	(0,74-1,92)	**3,05**	**(1,93-4,83)**
Eltern von Kindern 6-17 J.	**0,62**	**(0,41-0,94)**	0,79	(0,52-1,20)	1,27	(0,83-1,92)
Keine Kinder <18 J.	**0,50**	**(0,40-0,62)**	**1,35**	**(1,10-1,66)**	**2,71**	**(2,19-3,37)**
Gesamt	**0,51**	**(0,43-0,62)**	**1,24**	**(1,04-1,48)**	**2,41**	**(2,01-2,88)**

Tabelle A4: Multivariate logistische Regression für die Belastung in verschiedenen Subgruppen von Eltern mit Kindern im Schulalter mit den Kovariablen Alter, Geschlecht, Schulbildung und Migrationshintergrund. OR: Odd Ratios. 95% KI: 95% Konfidenzintervall. Signifikante Ergebnisse sind fett gedruckt.

	Welle 5 (31. März/1. April 2020)			Welle 15 (23./24. Juni 2020)			Welle 34 (26./27. Januar 2021)		
	Belastung = "Ja" (%)	OR	95% KI	Belastung = "Ja" (%)	OR	95% KI	Belastung = "Ja" (%)	OR	95% KI
Gesamt	58,5%			46,7%			52,6%		
**Alter (kontinuierlich)**		1,00	(0,96-1,03)		1,02	(0,98-1,05)		1,00	(0,97-1,03)
**Geschlecht**									
Männlich (Ref.)	56,0%	-	-	49,4 %	-	-	45,9 %	-	-
Weiblich	61,4%	1,20	(0,66-2,18)	44,2 %	0,79	(0,44-1,43)	59,3 %	1,70	(0,92-3,16)
**Dauer der Schulbildung**									
Bis zu 9 Jahren	57,1%	0,90	(0,30-2,82)	38,5 %	0,69	(0,21-2,30)	50,0 %	0,79	(0,25-2,54)
Mind. 10 Jahre, ohne Abitur	61,5%	1,17	(0,60-2,28)	50,7 %	1,30	(0,69-2,44)	50,9 %	0,87	(0,44-1,70)
Abitur (Ref.)	57,4 %	-	-	45,2 %	-	-	53,9 %	-	-
**Migrationshintergrund**									
Bekannt	68,8%	1,72	(0,76-3,91)	61,3 %	**2,34**	**(1,03-5,34)**	69,2 %	2,20	(0,89-5,42)
Nicht bekannt (Ref.)	56,8%	-	-	43,8 %	-	-	49,7 %	-	-
**Pseudo-R^2**			0,018			0,042			0,050

Tabelle A5: Vergleich elternspezifischer Belastungen zwischen Müttern und Vätern von Kindern im Schulalter in Welle 12 durch den Mann-Whitney-U-Test. Die Zahl der gültigen Fälle variiert für die letzteren acht Variablen, da nicht alle Aspekte von Elternschaft auf alle Teilnehmenden zutreffen. Signifikante Ergebnisse (p<0.05) sind fett gedruckt.

Welle 12 (19./20. Mai 2020)<br>Variablen (Kurzbeschreibung)	Gültige Fälle (n)	Mann-Whitney-U-Test	Z	asymp. p-Wert	Korrelationskoef. r
Ich fühle mich als Elternteil in der aktuellen Situation überfordert.	184	3438,5	-2,229	**0,03**	0,16
Die aktuelle Situation bringt unsere Familie an den Rand ihrer Kräfte.	184	4126,5	-0,291	0,77	0,02
Home-Office und Kinderbetreuung in Einklang zu bringen	113	1181,5	-2,411	**0,02**	0,23
Das Kind/die Kinder ohne den Kontakt zu anderen Kindern bei Laune zu halten	166	2453	-3,241	**<0,01**	0,25
Das Haus weniger verlassen zu können und Abstand zu halten	167	2826,5	-2,136	**0,03**	0,17
Die schulische Bildung des Kindes/der Kinder zu Hause zu organisieren.	163	2342	-3,297	**<0,01**	0,26
Jedem Kind eigene Zeit und eigenen Raum zu ermöglichen	151	2229	-2,349	**0,02**	0,19
Die Großeltern nicht mehr sehen zu können	144	1765,5	-3,356	**<0,01**	0,28
Auf familiäre und andere Hilfe im Familienleben (z.B. Babysitter) verzichten zu müssen.	119	1220,5	-2,927	**<0,01**	0,27
Mir mit meinem Partner/meiner Partnerin in Bezug auf die Kindererziehung einig zu sein.	148	2441,5	-1,112	0,27	0,09

Tabelle A6: Vergleich elternspezifischer Belastungen zwischen Müttern und Vätern von Kindern im Schulalter in Welle 30 durch den Mann-Whitney-U-Test. Die Zahl der gültigen Fälle variiert für die letzteren acht Variablen, da nicht alle Aspekte von Elternschaft auf alle Teilnehmenden zutreffen. Signifikante Ergebnisse (p<0.05) sind fett gedruckt.

Welle 30 (15./16. Dezember 2020)<br>Variablen (Kurzbeschreibung)	Gültige Fälle (n)	Mann-Whitney-U-Test	Z	asympt. p-Wert	Korrelationskoef. r
Ich fühle mich als Elternteil in der aktuellen Situation überfordert.	188	3558,0	-2,34	**0,02**	0,17
Die aktuelle Situation bringt unsere Familie an den Rand ihrer Kräfte.	188	3648,5	-2,09	**0,04**	0,15
Home-Office und Kinderbetreuung in Einklang zu bringen.	122	1574,5	-1,49	0,14	0,13
Das Kind/die Kinder ohne den Kontakt zu anderen Kindern bei Laune zu halten.	165	2575,0	-2,73	**0,01**	0,21
Das Haus weniger verlassen zu können und Abstand zu halten.	167	2736,5	-2,43	**0,02**	0,19
Die schulische Bildung des Kindes/der Kinder zu Hause zu organisieren.	165	2510,5	-2,95	**<0,01**	0,23
Jedem Kind eigene Zeit und eigenen Raum zu ermöglichen.	161	2792,0	-1,53	0,13	0,12
Die Großeltern nicht mehr sehen zu können.	148	2170,5	-2,20	**0,03**	0,18
Auf familiäre und andere Hilfe im Familienleben (z.B. Babysitter) verzichten zu müssen.	120	1411,5	-2,07	**0,04**	0,19
Mir mit meinem Partner/meiner Partnerin in Bezug auf die Kindererziehung einig zu sein.	142	2454,0	-0,257	0,80	0,02
