# Supplementary material for: Burden in parents of school-aged children during different phases of the COVID-19 pandemic in Germany: an analysis within the COVID-19 snapshot monitoring (COSMO) study
Source: Bundesgesundheitsblatt Gesundheitsforschung Gesundheitsschutz. 2021 Nov 26;64(12):1500–11. [Article in German] doi: 10.1007/s00103-021-03453-3 (PMC8617549; doi:10.1007/s00103-021-03453-3)
Supplement: Supplementary file 2 [file 103_2021_3453_MOESM2_ESM.pdf]

Burden in parents of school-aged children during different phases of the COVID-19 pandemic in Germany: an analysis within the COVID-19 snapshot monitoring (COSMO) study

Belastung von Eltern mit Kindern im Schulalter während verschiedener Phasen der COVID-19-Pandemie in Deutschland: Eine Analyse der COVID-19 Snapshot Monitoring (COSMO) Daten

Julia Elisabeth Rabe[1,2,3], Hannah Schillok[1,2], Christina Merkel[4], Stephan Voss[1,2], Michaela Coenen[1,2], Freia De Bock[4], Ursula von Rüden[4], Anke Bramesfeld[3], Caroline Jung-Sievers[1,2] and on behalf of the COSMO group[5]

[1]Institute for Medical Information Processing, Biometry and Epidemiology, Chair of Public Health and Health Services Research, LMU Munich, Elisabeth-Winterhalter-Weg 6, 81377 Munich, Germany.

[2]Pettenkofer School of Public Health, Elisabeth-Winterhalter-Weg 6, 81377 Munich, Germany.

[3]Institute for Epidemiology, Social Medicine and Health System Research, Hanover Medical School, Carl-Neuberg-Straße 1, 30625 Hanover, Germany.

[4] Federal Centre for Health Education (BZgA), Maarweg 149-161, 50825 Cologne, Germany

[5]The COSMO group members are listed in the acknowledgements.

Abstract

BACKGROUND Parents face a variety of personal challenges during the COVID-19 pandemic while simultaneously being confronted with additional, school related pandemic containment measures. OBJECTIVES To investigate burden in parents of school-aged children across different phases of the COVID-19 pandemic in Germany and to identify particularly affected subgroups. METHODS The COSMO project is a repetitive cross-sectional survey monitoring the psychosocial situation of the population in Germany during the pandemic with a sample size of approx. n=1000 respondents per survey wave. A quantitative analysis of COSMO data was conducted, using the item "burden" as main outcome, and, if applicable, on parenthood-related burden from March 2020 until January 2021. RESULTS During the first COVID-19 wave, parents of school-aged children were significantly more burdened compared to the general study population. However, burden decreased significantly from March/April to June 2020. During the second COVID-19 wave in January 2021, burden was homogeneously high across all groups. Single parenthood, a low household income, having a chronic health condition, a COVID-19 infection and a migration background were associated with higher burden, although none of these factors was consistently significant across the survey waves. Mothers reported to be more affected by parenthood-related burden than fathers. CONCLUSIONS School measures for infection control have to be weighed carefully against the psychological impact on parental burden with subsequent negative impact on the family system.

Key words: Mental health, stress, school, pandemic, containment measures

1. Introduction

The surge of COVID-19 infections in March 2020 led to comprehensive non-pharmaceutical containment measures in Germany, as in most countries around the globe. These included wearing masks, repeated contact restrictions, testing and quarantining regimentations as well as closure of large parts of public life. In comparison to other population groups, children and their caregivers saw themselves confronted with an additional, i.e., school-related set of measures. According to data from the UNESCO education response panel, on the 1st of April 2020, 173 countries had installed nationwide school closures, affecting 84.3% of total enrolled learners across all educational stages, and thus also their parents[1]. Learning continued either through distant learning, home-schooling administered by parents, or no schooling at all.

During these difficult times, parents faced a variety of challenges: caring for their own health and mental well-being, maintaining their professional life during pandemic restrictions, organising their children's schooling - without the support of grandparents or other domestic help - and attending to their children's needs for emotional support. The latter had increased, by the COVID-19 pandemic in general[2–4] and by school closures in particular through the loss of social contacts as well as the loss of opportunities for education, physical activities and play[5].

From past epidemics it is known that such a constellation puts family members, both parents and their children, at risk for mental health problems: a study investigating H1N1, SARS and avian influenza found isolated or quarantined parents and children at elevated risk of posttraumatic stress disorder [PTSD]. Parental and infant mental health were closely related: among parents at risk of PTSD, 87.5% children displayed according symptoms, too[6].

This interconnectedness of parental and infant mental health is based on the bonding between parents and children within a family system, that is important for a healthy development in children in general[7, 8]. Therefore, when concerned about the health of children during the pandemic, we must keep the health of parents in mind, too. However, parents have been less extensively researched during the COVID-19 pandemic than children so far. First national[9–11] and international[12–18] results indicate that parents are particularly affected by the pandemic measures.

As the administration of containment measures varied over the first months of the pandemic, levels of anxiety, depression and distress in the general population in Germany changed, too[19]. However, data on changes in the situation of parents with different bundles of hardships at different time points is still scarce.
Therefore, the objective of this study was threefold: with the unique ability of COSMO data to tightly monitor the burden of parents across different phases of the pandemic, we aim to analyse parental burden in relation to different pandemic phases and context factors. Secondly, we aim to identify especially burdened subgroups within the group of parents that might require extended support. Thirdly, we aim to understand which aspects of the changed parenting conditions were perceived as particularly burdensome, to potentially being able to alleviate these aspects in a next pandemic phase.

2. Methods

2.1. Study sample – the COSMO project

The COVID-19 snapshot monitoring (COSMO) project is a repetitive cross-sectional study aiming to capture the broad psychosocial status of the German population during the pandemic. In weekly to bi-weekly turns (called "waves"), starting in March 2020, approximately n = 1000 people aged 18 to 74

are being questioned about their individual psychosocial situation, their knowledge about COVID-19 as well as their attitudes towards several institutions, authorities and measures to contain the pandemic.
The project uses a non-probability quota sample and aims at a representative distribution of the participants in terms of age × gender and federal states. Participants are recruited by the online access panel of the market and social research company ‘respondi’ and receive remuneration for their participation[20].
COSMO is a joint project by the University of Erfurt, the Robert Koch Institute, the German Federal Centre for Health Education (“Bundeszentrale für gesundheitliche Aufklärung”), the Leibniz Institute for Psychology, the Science Media Centre, the Bernhard-Nocht-Institute for Tropical Medicine, and the Yale Institute for Global Health[21]. While some items are collected constantly, most variables change over the course of the project and are therefore collected irregularly in order to adapt to the societal changes as the pandemic unfolds. Further details are described in the study protocol[22]. The project however has evolved since its publication.

2.2. Evaluation time points

Up to this point, the COVID-19 pandemic in Germany is characterized by different phases that occur as waves. The first COVID-19 wave was observed from early March 2020 to mid-May 2020. The second COVID-19 wave followed at the end of September and lasted until the end of February 2021[23]. To analyse mental health challenges over the course of the pandemic, we chose the COSMO assessment waves 5 (31st of March/ 1st of April 2020), 15 (23rd/24th of June 2020) and 34 (26th/27th of January 2021) for the main outcome variable burden (see Figure 1).

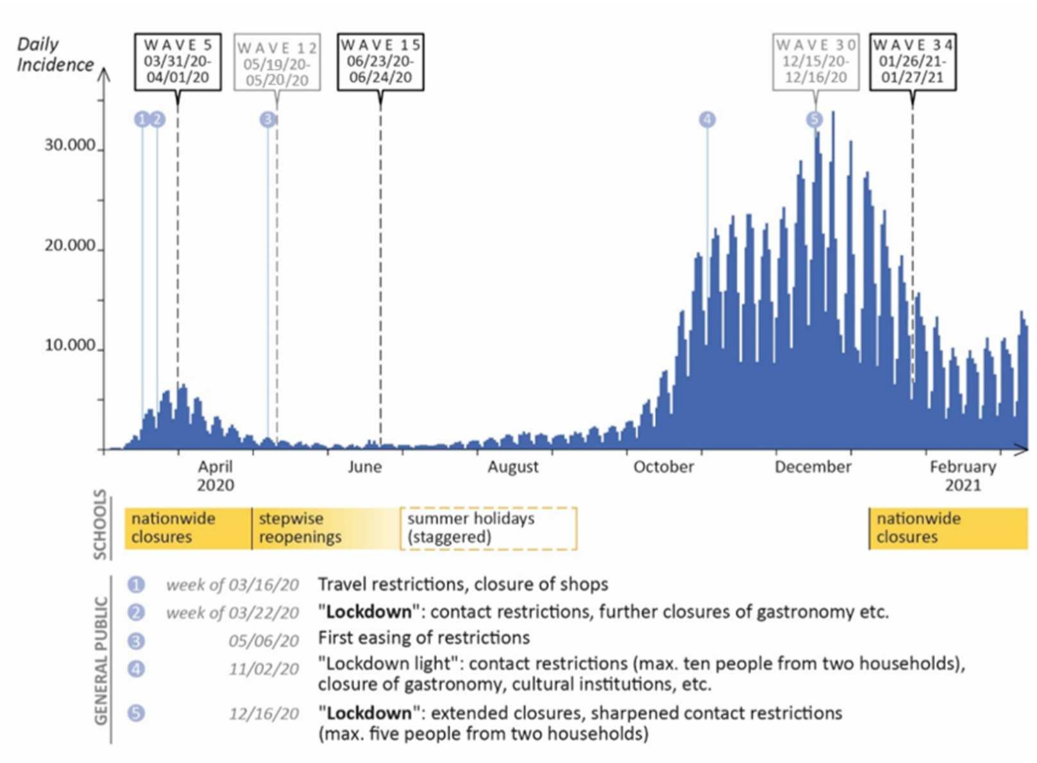

Figure 1 – Development of the pandemic in Germany and evaluation time points of the COSMO study for the three main analyses (black) and two sub-analyses (grey) presented here. Development of COVID-19 incidences and important containment measures for the general population and schools in Germany from March 2020 to February 2021. Own illustration based on incidence data from the RKI Dashboard[38].

The dates chosen represent time points when all federal states in Germany had similar regimentations on the operation of schools in place: COSMO wave 5 was assessed during the first "lockdown" beginning in mid-March 2020[24]. COSMO wave 15 represents a short time span in which all schools had generally returned from full school closures to in-person teaching under various containment measures, with only one state (Mecklenburg – West Pomerania) already being in summer term break for one day[25, 26]. Schools were re-closed in all federal states in the week of December 14th 2020 with consecutive distant learning in place[27]. COSMO wave 34 captures the situation.
At any point of school closures, there was emergency day care available for children whose parents worked in jobs that were considered critical infrastructure.
For additional analyses, we analysed a set of questions on parenthood-related burden in COSMO wave 12 (May 19th/20th2020) and 30 (December 15th/16th 2020) that was only assessed at these two dates. Wave 12 represents the situation during the stepwise opening phase after the first COVID-19 wave in Germany. COSMO Wave 30 was assessed at the time point directly after the announcement of full nationwide school re-closures in December 2020.

2.3. Variables and Measures

Main outcome

As main outcome, we used the variable "burden" by survey questions where participants were asked whether they find their overall current personal situation to be burdensome or not (yes/no, binary).

Inclusion criteria and demographic co-variables

In temporal alignment with the current S3 guideline on prevention and control of SARS-CoV-2 transmission in schools, we focus here on the effects on parents/caregivers from school interventions or closures[28]. Therefore, we consider the burden on parents of minor, potentially school-aged children aged 6-17 years alongside descriptive data from parents of younger children (0-5 years) or adult children/respondents without any children.

Included in the analyses were gender and age of participants as well as ages of the respective children. Additionally, participating parents were asked whether they are single parents.

Socioeconomic co-variables

To assess participants' educational background, respondents were asked about the duration of school education they have received. Participants were asked about their household size and whether they have a migration background. As of wave 15, the participants' employment status and their net household income were assessed.

2.3.4 Health related co-variables

Participants were asked whether they have a chronic condition, whether they allocate themselves to the risk group for COVID-19 infections (as of wave 15) and whether they have or have had a COVID-19 infection.[a]

2.3.5 Variables assessing parenthood-related burden

In order to characterise which aspects of parenting during the COVID-19 pandemic were perceived as particularly burdensome, we used the COSMO variable set of parenthood-related burden for additional analyses. This set was collected in wave 12 (May 2020) and 30 (December 2020) only and comprised two modes of questioning. Firstly, parenthood-related burden was assessed by the two

[a] Here, the mode of data collection has changed over course of the project (COSMO waves 5 and 15: "Yes, diagnosis confirmed", "Yes, diagnosis not yet confirmed", "Yes, convalesced", "No" and "Don't know", wave 34: "Yes", "No").

statements a) “I feel overwhelmed as a parent by the current situation” and b) “The current situation brings our family to the edge of our forces[b]”. Secondly, COSMO asked parents how challenging they found eight specific aspects of parenting during the pandemic.

2.4. Statistical Analysis

We grouped participants into i) parents of school-aged children as our main group of interest, ii) parents of children younger than school-age and iii) adults without underage children or without any children. To analyse the development of general burden in parents of school-aged children across the three COSMO-waves 5, 15 and 34 in comparison to the other groups, we calculated absolute and relative frequencies of participants feeling burdened for each group and wave. We used Cramer’s V and reported p-values to analyse the distribution of burden per wave cross-sectionally. For the comparison across time, we calculated Odds Ratios (ORs) and corresponding 95% confidence intervals (95% CIs). In order to estimate whether our chosen survey dates were representative for the respective phase of the pandemic, we conducted a sensitivity analysis in which we compared perceived burden between parents and the general study sample at all time points this variable was collected at.
In order to identify particularly affected subgroups within the parents of school-aged children, we calculated descriptive statistics for the aforementioned co-variates and performed a univariate logistic regression. Based on publications by Ravens-Siever in the COPSY study, the variables age, gender, educational background and migration background of the parents were included in the model for a multivariate logistic regression[4]. We report ORs, 95% CIs and Nagelkerke’s R. Goodness-of-fit was controlled applying the Hosmer-Lemeshow-Test.
In order to explore parenthood-related burden in parents of school-aged children within an additional analysis, we calculated descriptive statistics for COSMO waves 12 and 30 and compared burden of mothers and fathers using Mann-Whitney-U-tests. The correlation coefficient r was used as a relevant measure of effect size.

For all calculations, we considered p-values <0.05 to be significant. Due to the exploratory nature of this study, we refrained from adjusting for multiple testing. All analyses were performed using SPSS, version 27.0 (IBM, Armonk, New York, United States).

3. Results

3.1. Sample characteristics

We present detailed study sample characteristics for the total study population and for the main group of parents of school-aged children in table 1. Characteristics of the group of parents with younger children and of adults without underage children or any children at all are shown in the online supplementary materials (Appendix S1).
Wave 5 comprised 1028 participants, including 188 parents of school-aged children, accounting for 18.3% of the total study population. Wave 15 and 34, with 993 and 1001 participants in total, had similar proportions of parents of school-aged children; 184 (18.5%) parents in wave 15 and 171 (17.1%) parents in wave 34. Parents counted themselves less often to the risk group for COVID-19, lived on average in larger households and had larger net household incomes.

[b] “Family” referred to the people the respondents share their household with.

Study sample description	Wave 5 (March 31st/April 1st 2020)				Wave 15 (June 23rd/24th 2020)				Wave 34 (January 26th/27th 2021)			
	Parents of children 6-17 Y.		All		Parents of children 6-17 Y.		All		Parents of children 6-17 Y.		All	
	n/mean	%/SD	n/mean	%/SD	n/mean	%/SD	n/mean	%/SD	n/mean	%/SD	n/mean	%/SD
Total	188	100.0%	1028	100.0 %	184	100.0%	993	100.0 %	171	100.0%	1001	100.0 %
Gender												
Male	100	53.2 %	507	49.3 %	89	48.4 %	483	48.6 %	85	49.7 %	504	50.3 %
Female	88	46.8 %	521	50.7 %	95	51.6 %	510	51.4 %	86	50.3 %	497	49.7 %
Age of participant (continuous, in years)	43,26	8.02	45.86	15,96	41.49	8.36	45.81	15.51	41.31	9.51	44.5	15.53
Age of participant (categorical)												
18-29 Y.	9	4.8 %	199	19.4 %	11	6.0 %	178	17.9 %	14	8.2 %	192	19.2 %
30-39 Y.	47	25.0 %	160	15.6 %	65	35.3 %	221	22.3 %	60	35.1 %	243	24.3 %
40-49 Y.	92	48.9 %	234	22.8 %	77	41.8 %	166	16.7 %	63	36.8 %	150	15.0 %
50+ Y.	40	21.3 %	435	42.3 %	31	16.8 %	428	43.1 %	34	19.9 %	416	41.6 %
Age of Children (several quotes possible)												
6-9 Y.	81	43.1 %	n/a	n/a	84	45.7 %	n/a	n/a	75	43.9 %	n/a	n/a
10-13 Y.	69	36.7 %	n/a	n/a	74	40.2 %	n/a	n/a	74	43.3 %	n/a	n/a
14-17 Y.	84	44.7 %	n/a	n/a	74	40.2 %	n/a	n/a	63	36.8 %	n/a	n/a
Single parent												
Yes	33	17.6 %	n/a	n/a	36	19.6 %	n/a	n/a	29	17.0 %	n/a	n/a
No	155	82.4 %	n/a	n/a	148	80.4 %	n/a	n/a	142	83.0 %	n/a	n/a
School education (SE)												
Up to 9 years of SE	14	7.4 %	104	10.1 %	13	7.1 %	112	11.3 %	14	8.2 %	119	11.9 %
At least 10 years of SE without A-Levels	52	27.7 %	360	35.0 %	67	36.4 %	340	34.2 %	55	32.2 %	309	30.9 %
At least 10 years of SE, graduated with A-Levels	122	64.9 %	564	54.9 %	104	56.5 %	541	54.5 %	102	59.6 %	573	57.2 %
Employment												
Yes	ND	ND	ND	ND	150	81.5 %	659	66.4 %	149	87.1 %	692	69.1 %
No	ND	ND	ND	ND	34	18.5 %	334	33.6 %	22	12.9 %	309	30.9 %
Net household income												
<1250€	ND	ND	ND	ND	8	4.3 %	142	14.3 %	8	4.7 %	115	11.5 %
1250-2249	ND	ND	ND	ND	32	17.4 %	249	25.1 %	27	15.8 %	245	24.5 %
2250-3999	ND	ND	ND	ND	88	47.8 %	352	35.4 %	83	48.5 %	367	36.7 %
4000+	ND	ND	ND	ND	45	24.5 %	170	17.1 %	46	26.9 %	199	19.9 %
Not answered	ND	ND	ND	ND	11	6.0 %	80	8.1 %	7	4.1 %	75	7.5 %
Migration background												
Yes	32	17.0 %	149	14.5 %	31	16.8 %	150	15.1 %	26	15.2 %	187	18.7 %
No	155	82.4 %	876	85.2 %	151	82.1 %	839	84.5 %	143	83.6 %	811	81.0 %
Don't know	1	0.5 %	3	0.3 %	2	1.1 %	4	0.4 %	2	1.2 %	3	0.3 %
Household size												
Just me	13	6.9 %	269	26.2 %	4	2.2 %	262	26.4 %	10	5.8 %	231	23.1 %
2 people	23	12.2 %	400	38.9 %	19	10.3 %	387	39.0 %	12	7.0 %	404	40.4 %
3-4 people	127	67.6 %	313	30.4 %	128	69.6 %	293	29.5 %	114	66.7 %	302	30.2 %
5 or more	25	13.3 %	46	4.5 %	33	17.9 %	51	5.1 %	35	20.5 %	61	6.1 %
Not answered	0	0 %	0	0 %	0	0 %	0	0 %	0	0 %	3	0.3 %

Chronic condition												
Yes	55	29.3 %	336	32.7 %	57	31.0 %	338	34.0 %	50	29.2 %	332	33.2 %
No	127	67.6 %	651	63.3 %	122	66.3 %	632	63.6 %	116	67.8 %	634	63.3 %
Don't know	6	3.2 %	41	4.0 %	5	2.7 %	23	2.3 %	5	2.9 %	35	3.5 %
Affiliation to risk group for COVID-19												
Yes	ND	ND	ND	ND	76	41.3 %	518	52.2 %	42	24.6 %	349	34.9 %
No	ND	ND	ND	ND	108	58.7 %	475	47.8 %	113	66.1 %	572	57.1 %
Don't know	ND	ND	ND	ND	0	0 %	0	0 %	16	9.4 %	80	8.0 %
Personal COVID-19 infection (5 and 15/34)[a]												
Yes confirmed / Yes	6	3.2%	9	0.9 %	1	0.5 %	6	0.6 %	13	7.6 %	56	5.6 %
Yes, not yet confirmed/ No	3	1.6%	11	1.1 %	4	2.2 %	11	1.1 %	158	92.4 %	945	94.4 %
No / -	159	84.6%	868	84.4 %	154	83.7 %	882	88.8 %	-	-	-	-
Yes, convalesced / -	ND	ND	ND	ND	7	3.8 %	10	1.0 %	-	-	-	-
Don't know / -	20	10.6%	140	13.6 %	18	9.8 %	84	8.5 %	-	-	-	-

Table 1 - Sociodemographic and health-related data for the entire sample as well as for parents with school-age children (6-17 years) for the COSMO survey waves of the main analysis 5, 15 and 34. ND: no data available; n.a.: not applicable; SD: standard deviation; *For this variable the mode of data collection has changed over course of the project (COSMO waves 5 and 15: "Yes, diagnosis confirmed", "Yes, diagnosis not yet confirmed", "Yes, convalesced", "No" and "Don't know", wave 34: "Yes", "No").*

3.2. Parental burden in different phases of the pandemic

Figure 2 shows the development of perceived burden of parents of school-aged children over all available time points in COSMO from wave 5 to 34. COSMO wave 5 captures well the initial level of burden, wave 15 the general trend of decrease. The lowest levels of burden however were found in July and September 2020, where burden of parents of school-aged children and of the general study population was almost identical. This coincided with summer holidays. Wave 34 represents well the increased levels of burden in the second COVID-19 wave in Germany, but is an exception in that parental burden levels usually laid somewhat above that of the general study population during the second COVID-19 wave. Downward spikes in the week after Easter and around Christmas 2020 indicate relief by holidays for both groups.

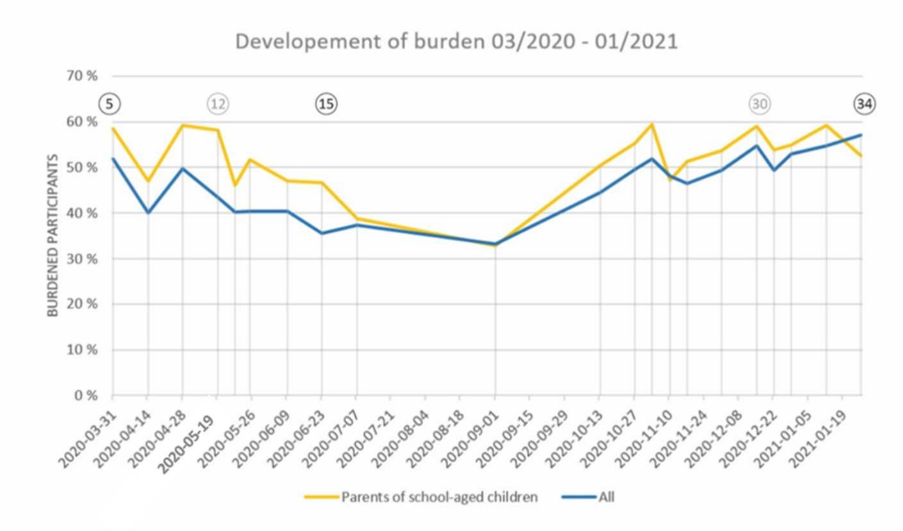

Figure 2 - Perceived burden of parents of school-aged children across all available survey points in COSMO from wave 5 to 34 in comparison to the general study population: The waves of our main analysis are indicated in black (wave 5, 15, 34), those of our additional analysis in grey (wave 12 and 30).

Figure 3 shows the proportion of study participants in COSMO waves 5, 15 and 34 who perceive their current situation as burdensome, stratified by subgroups.

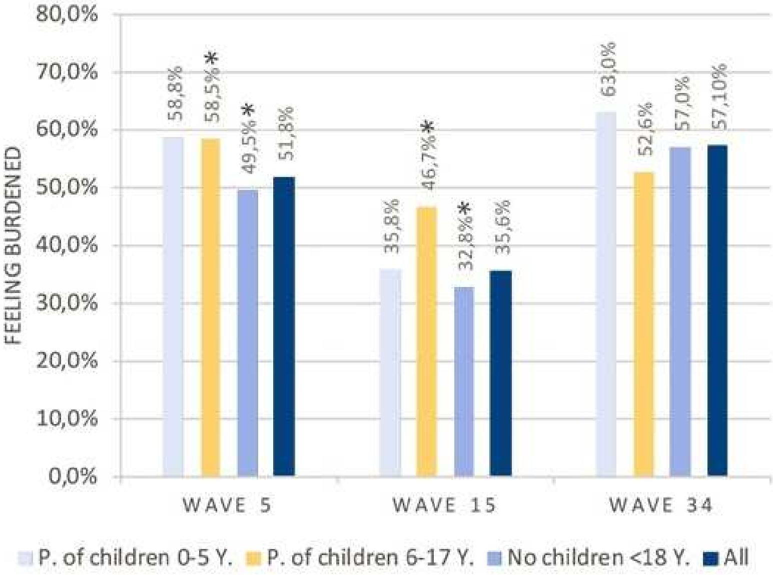

Figure 3– Perceived burden stratified by subgroups in COSMO wave 5 (March/April 2020), 15 (June 2020) and 34 (January 2021). Parents can have both pre-school-aged and school-aged children at the same time. Significant results from the cross-sectional comparison within each wave are marked with "".*

For the general study population, the number of burdened people decreased from the first COVID-19 wave in March/April to June 2020 and increased with the second COVID-19 wave to higher levels than during the first COVID-19 wave (51.8% vs. 35.6% vs. 57.1%). All observed changes were statistically significant.

For parents of school-aged children, this pattern was similar with a significant decrease from March to June 2020 (wave 5 vs. wave 15). However, there was no significant surge from wave 15 to wave 34.

In the cross-sectional comparison of the groups, parents of school-aged children felt significantly more often burdened than the general study population in wave 5 and 15 (58.5% and 46.7% respectively, compared to 51.8% and 35.6% in the total study population). In wave 34, parents with school-aged children form the least burdened group (52.6% compared to 57.1% in total). However, in this wave in January 2021 Cramer's V was not significant for any group. Further detailed results tables are shown in the online supplementary materials (S2 and S3).

3.3. Parental burden in different subgroups

Table 2 presents the relative proportion of parents with school-aged children feeling burdened per subgroup, as well as the results from our univariate logistic regression analysis.

	Wave 5 (March 31st/April 1st 2020)			Wave 15 (June 23rd/24th 2020)			Wave 34 (January 26th/27th 2021)		
	Burden = "Yes" (%)	OR	95% CI	Burden = "Yes" (%)	OR	95% CI	Burden = "Yes" (%)	OR	95% CI
Gender									
Male (reference)	56.0 %	-	-	49.4 %	-	-	45.9 %	-	-
Female	61.4 %	1.25	(0.70-2.24)	44.2 %	0.81	(0.45-1.45)	59.3 %	1.72	(0.94-3.15)
Age of participants									
18-29 Y.	44.4 %	0.59	(0.14-2.54)	54.5 %	1.13	(0.28-4.47)	28.6 %	0.40	(0.11-1.53)
30-39 Y.	66.0 %	1.43	(0.60-3.42)	36.9 %	0.55	(0.23-1.31)	56.7 %	1.31	(0.56-3.04)
40-49 Y.	56.5 %	0.96	(0.45-2.04)	51.9 %	1.10	(0.44-2.33)	55.6 %	1.25	(0.54-2.88)
50+ (reference)	57.5 %	-	-	51.6 %	-	-	50.0 %	-	-
Age of Children (several quotes possible)[a]									
6-9 Y.	61.7 %	1.26	(0.70-2.28)	48.8 %	1.17	(0.65-2.09)	53.3 %	1.05	(0.57-1.93)
10-13 Y.	62.3 %	1.28	(0.70-2.36)	40.5 %	0.66	(0.36-1.19)	48.6 %	0.75	(0.41-1.38)
14-17 Y.	52.4 %	0.63	(0.35-1.14)	54.1 %	1.64	(0.90-2.96)	49.2 %	0.81	(0.43-1.50)
Single parent									
Yes	66.7 %	1.52	(0.69-3.36)	63.9 %	2.39	(1.12-5.07)	69.0 %	2.29	(0.97-5.36)
No (reference)	56.8 %	-	-	42.6 %	-	-	49.3 %	-	-
School education (SE)									
Up to 9 years of SE	57.1 %	0.99	(0.32-3.03)	38.5 %	0.76	(0.23-2.47)	50.0 %	0.86	(0.28-2.61)
At least 10 years of SE, without A-Levels	61.5 %	1.19	(0.61-2.31)	50.7 %	1.25	(0.68-2.31)	50.9 %	0.89	(0.46-1.71)
A-Levels (reference)	57.4 %	-	-	45.2 %	-	-	53.9 %	-	-
Employment									
Yes (reference)	ND	ND	ND	45.3 %	-	-	54.4 %	-	-
No	ND	ND	ND	52.9 %	1.36	(0.64-2.86)	40.9 %	0.58	(0.23-1.44)
Net household income (collapsed)									
<1250€	ND	ND	ND	87.5 %	12.69	(1.43-112.51)	25.0 %	0.43	(0.08-2.38)
1250-2249	ND	ND	ND	53.1 %	2.05	(0.82-5.18)	51.9 %	1.40	(0.54-3.63)
2250-3999	ND	ND	ND	46.6 %	1.58	(0.75-3.32)	57.8 %	1.78	(0.86-3.69)
4000+ (reference)	ND	ND	ND	35.6 %	-	-	43.5 %	-	-
Migration background									
Yes	68.8 %	1.70	(0.76-3.83)	61.3 %	2.03	(0.92-4.48)	69.2 %	2.28	(0.93-5.58)
No/Don't know (reference)	56.8 %	-	-	43.8 %	-	-	49.7 %	-	-
Household size									
Just me (reference)	53.8 %	-	-	50.0 %	-	-	40.0 %	-	-
2 people	60.9 %	1.33	(0.34-5.27)	63.2 %	1.71	(0.20-15.02)	41.7 %	1.07	(0.19-5.91)
3-4 people	56.7 %	1.12	(0.36-3.53)	43.8 %	0.78	(0.11-5.70)	57.0 %	1.99	(0.53-7.44)
5 or more	68.0 %	1.82	(0.46-7.22)	48.5 %	0.94	(0.12-7.50)	45.7 %	1.26	(0.30-5.28)
Not answered									
Chronic condition (of parents)									
Yes	65.5 %	1.51	(0.79-2.90)	63.2 %	2.64	(1.39-5.03)	58.0 %	1.36	(0.70-2.64)
No/Don't know (reference)	56.7 %	-	-	39.4 %	-	-	50.4 %	-	-

Affiliation to risk group for COVID-19 (of parents)									
Yes	ND	ND	ND	55.3 %	1.80	(0.99-3.25)	52.4 %	0.99	(0.49-1.98)
No/Don't know (reference)	ND	ND	ND	40.7 %	-	-	52.7 %	-	-
Personal COVID-19 infection (5 and 15/34)[b]									
Yes confirmed / Yes	66.7 %	X	X	100.0 %	X	X	84.6 %	5.50	(1.18-25.62)
Yes, not yet confirmed/ No	100.0 %	X	X	75.0 %	X	X	50.0 %	-	-
No / -	57.2 %	X	X	44.8 %	X	X	ND	ND	ND
Yes, convalesced / -	-	X	X	42.9 %	X	X	ND	ND	ND
Don't know / -	60.0 %	X	X	55.6 %	X	X	ND	ND	ND

Table 2 -Subjective perceived burden of parents of school-aged children, univariate binary logistic regression analysis, including relative frequency of perceived burden, odds ratios (OR) and 95% confidence intervals (95% CI). Significant results are printed in bold. ND: not collected. X: Due to very small subgroups, a regression analysis was not performed. [a.] *Since this variable was collected as multiple response item, respondents were able to select more than one answer option for this variable (multiple quotes possible). Therefore, a reference category was not defined for calculating the OR; instead, the group that chose this answer option ("quoted") was compared with the group that did not choose this answer option ("not quoted").* [b.] *For this variable the mode of data collection has changed over course of the project (COSMO waves 5 and 15: "Yes, diagnosis confirmed", "Yes, diagnosis not yet confirmed", "Yes, convalesced", "No" and "Don't know", wave 34: "Yes", "No").*

More mothers than fathers felt burdened during the first COVID-19 wave in late March/early April 2020 (56.0% vs 61.4%) and during the second wave in January 2021 (45.9% vs 59.3%), whereas in June 2020, fathers were more often burdened than mothers (49.4% vs. 44.2%). However, tests failed to be significant. We could not find any correlation between the age of parents or their children with regard to an increased perceived burden.

Single parenthood was significantly associated with feeling burdened in wave 15 in June 2020 (OR 2.39, 95% CI 1.12-5.07). This pattern was found in wave 5 and 34, too, but was not significant then. For socioeconomic variables, only a very low household income below 1250€ per month was significantly associated with higher rates of burden in wave 15 (OR 12.69, 95% CI 1.43-112.51). While parents with a migration background quoted to be burdened more often across all waves, none of the results were statistically significant.

Concerning health-related covariates, having a chronic condition was associated with being burdened more often across all waves (65.5% vs 56.7% in wave 5, 63.2% vs 39.4% in wave 15 and 58.0% vs. 50.4% in wave 34), albeit only in wave 15 the difference was statistically significant (OR 2.64, 95% CI 1.39-5.03). For the group of parents pertaining to the risk group for COVID-19 we obtained no significant results. A previous COVID-19 infection was significantly associated with feeling burdened in wave 34 (84.6% vs 50%, OR 5.50, 95% CI 1.18-25.62).

According to our multivariate regression (online supplementary material S4), in wave 15 having a migration background was significantly associated with more prevalent burden (OR 2.34, 95% CI 1,03-5,34). Age, school education, and gender remained insignificant. Nagelkerke's Pseudo-R^2 was modest at all time points.

3.4 Analysis on specific aspects contributing to parental burden

COSMO wave 12 (May 2020) comprised n=184 parents of school-aged children, including 90 fathers (48.9%) and 94 mothers (51.1%), COSMO wave 30 (December 2020) n=188 parents of school-aged children, 95 fathers (50.5%) and 93 mothers (49.5%). Online supplementary material S5 shows boxplot diagrams for each item of the parenthood-related burden variable set for both waves. Reasons for

burden were quite consistent across both waves. Keeping the children happy without them having contact to their peers (Median 4.5 in wave 12, 5 in wave 30), organising the school education (Median 5) and not being able to see the grandparents anymore (Median 5) was perceived as most challenging.

Figure 4 presents boxplot diagrams for the same item set in wave 12, stratified for gender and shows a predominantly significant higher burden for mothers than for fathers. In the online supplementary materials, we report the results from the Mann-Whitney-U-Test (Table S5), together with the respective results for wave 30 (Table S6, Figure S2), that largely support this finding.

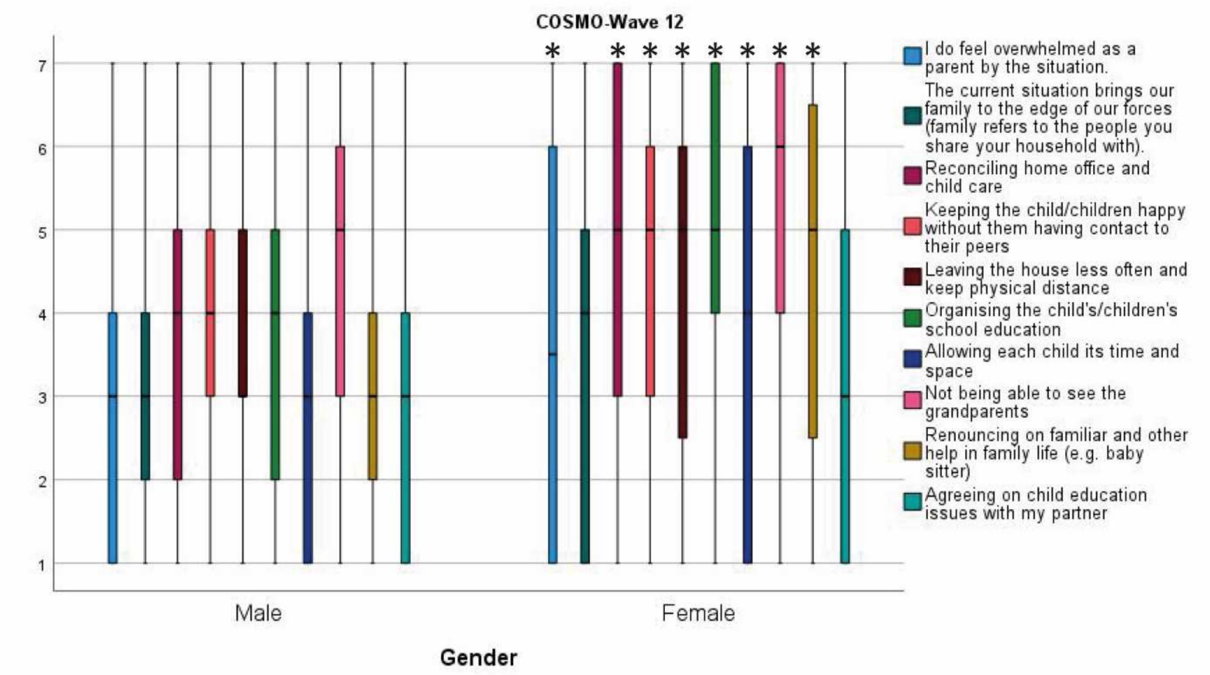

Figure 2 - Comparison of parenthood-related burden stratified by gender for wave 12. Boxplot diagrams for the parental burden variable set, comparing mothers to fathers of school-aged children in wave 12 (May 2020). Response options were 1 "Doesn't apply at all" to 7 "Fully applies" for the first two items, and 1 "Not at all challenging" to 7 "Extremely challenging" for the latter. Significant results from the Mann-Whitney-U-Test are marked with "".*

4. Discussion

In this study, we investigated the burden of parents at well-defined time points during the COVID-19 pandemic in Germany. Our most relevant findings were that parents of school-aged children were significantly more burdened during the first COVID-19 wave and the subsequent easing than the general study population. Mothers seemed to be more burdened by parenting under pandemic circumstances than fathers.

Our results are congruent with other studies on the situation of parents in Germany so far. Rothe et al. found higher perceived stress in parents than in adults not living with underage children in April and early May 2020[10]. Huebener et al. reported a stronger decrease in satisfaction with overall life, family life and childcare in parents of children under the age of 11 years than in adults without children in May and June 2020[11]. Calvano et al. found parents to be more stressed than before the pandemic [9].

International evidence from non-European countries confirm that increased parental stress and burden is a phenomenon that occurs worldwide during the COVID-19 pandemic (e.g., in the U.K. [15],

in the U.S.[12–14], in Italy[16, 17] and in Guatemala[18]), while higher stress levels are associated with younger age of parents and children.

We were able to identify the following aspects as risk factors for a higher parental burden: a chronic illness, low household income, single parent status, a COVID-19 infection and a migration background.

These factors were also mentioned in studies with similar research interest: German studies found having a mental health condition (as important subgroup of chronic conditions) to correlate with parental stress [10], just as a low household income[11]. Single parenthood was also correlated with parental stress and exhaustion in international studies[16, 17]. The results of the COPSY study showed that low parental educational attainment and parental migration background are associated with negative child mental health outcomes during the COVID-19 pandemic and will therefore also be relevant when assessing risk factors for parental stress[4].

With regard to the parent-specific burden of home schooling, our analyses showed that organising school education was perceived as one of the main challenges, also through not seeing the grandparents and omission of household help. In another German study, parents indicated distancing, restriction and closed childcare facilities to be most stressful[9]. Thorell et al. reported that provision of online teaching was limited in many countries including Germany, transmitting the main responsibility for schooling to parents[29]. The authors found increased parental stress in 57.2% of German parents due to home schooling, with even higher stress levels for parents with children with mental health conditions. German parents were similarly burdened by home schooling as parents in Italy, Spain, the U.K. and Belgium. Generally, parents across all countries report an increase in domestic conflicts[29].

However, a smaller number of parents also mention positive changes coming with the pandemic such as more family time and increased gratitude[9].

In our additional analysis of parenthood-related burden, questioned in May (wave 12) and December (wave 30) 2020, we observed in both waves a similar pattern of most challenging aspects, whereas mothers were more affected than their male counterparts. This suggests that during the pandemic the mental health of mothers is particularly at risk. Other publications also observed a higher decrease in life satisfaction for mothers than for fathers[11, 30]. This might not only have a potentially unfavourable effect on their health but also on their social and economic equality, as reported by several authors[11, 14, 16, 31]. Mothers with underage children reduced more hours of paid labour and took over disproportionally more of the additional care work during the COVID-19 pandemic. This has been observed in Germany[30, 32, 33] as in other countries such as the U.S.[34] and the U.K.[15, 35, 36]. In a British study, the only case in which mothers and fathers did the same amount of childcare was when mothers simultaneously pursued paid work and fathers did not. Additionally, employed mothers reported larger shares of interrupted paid work than their male counterparts[36]. For the U.S., however, Russel et al., identified fathers to be more stressed than mothers[12].

It is important to note that, due to the topicality of the issue, many of the studies mentioned were conducted using convenience sampling methods. Thus, their findings are therefore not based on representative samples, which could limit their validity.

Limitations

While the total sample of the COSMO study is representative in size with approx. n=1000 participants per survey wave, our relevant subgroup, parents of school-age children, was significantly smaller in every wave, which is why an overestimation or underestimation of the identified correlations cannot be ruled out. Since COSMO aims to capture snapshots on a broad range of topics rather than an in-

depth analysis of certain aspects, lengthy questionnaires or standardized diagnostic instruments could not be integrated. In addition, children-related data other than age was not included in COSMO.

There are also some limitations arising from COSMO's online-based data collection procedure that may compromise some of our findings: In COSMO, participants from lower socioeconomic groups are generally underrepresented while participants from higher socio-economic groups are clearly overrepresented and therefore influencing our results. As gender is collected binary only, other gender identities are not represented. Also, if a family is at the edge of their forces by their current situation, they might be less likely to take part in a scientific survey than those who are not. Thus, this might lead to a bias towards underestimation of burden.

5. Conclusion

This study indicates that especially parents were and are vulnerable to mental burden during different phases of the COVID-19 pandemic in Germany. It is important that parental burden is considered when deciding politically on pandemic containment measures. In addition, parents should be especially considered when tailoring offers for psychological and practical support. For instance, the World Health Organization (WHO) for the European Region suggests the development of target-group, needs-based education and support services[37], which could particularly address parents from risk factor loaded social backgrounds. Given the unequal burden of the pandemic on mothers and fathers, it is important that mothers are represented in policymaking panels and also receive tailored support.

Acknowledgements:

The COSMO group comprises Cornelia Betsch, Lars Korn, Lisa Felgendreff, Sarah Eitze, Philipp Schmid, Philipp Sprengholz (University of Erfurt, Nordhäuser Straße 63, 99089 Erfurt, Germany), Lothar H Wieler, Patrick Schmich (Robert Koch-Institute, Nordufer 20, 13353 Berlin, Germany), Heidrun Thaiss, Freia De Bock, Ursula von Rüden, Christina Merkel, Boris Orth (Federal Centre for Health Education, Maarweg 149-161, 50825 Cologne, Germany), Volker Stollorz (Science Media Center Germany gGmbH, Rosenstr. 42–44, 50678 Cologne, Germany), Michael Ramharter (Bernhard-Nocht-Institute for Tropical Medicine, Bernhard-Nocht-Straße 74, 20359 Hamburg, Germany), Michael Bosnjak (Leibniz Institute for Psychology Information, Universitätsring 15, 54296 Trier, Germany), Saad B. Omer (Yale Institute for Global Health, 1 Church Street, New Haven, CT, 06510, USA), Katrine Habersaat, Martha Scherzer and Cristiana Salvi (WHO Regional Office for Europe, Marmorvej 51, DK-2100 Copenhagen OE, Denmark).

Julia Elisabeth Rabe would like to thank Lina Bornemann for her valuable support with the illustration of the figures.

Conflict of Interests:

Julia Elisabeth Rabe, Hannah Schillok, Christina Merkel, Stephan Voss, Michaela Coenen, Freia De Bock, Ursula von Rüden, Anke Bramesfeld und Caroline Jung-Sievers declare no competing interests. No studies on humans or animals were performed by the authors for this article. For the studies listed, the ethical guidelines stated there apply respectively. Participation of the respondents in the interviews was voluntary. Respondents were informed that they were participating in a scientific study. The data records of all surveys were provided in anonymized form.

6. Appendix

6.1 Supplementary tables and figures

S1: Sociodemographic and health-related data for parents with children aged 0 to 5 years and for participants without minor children in waves 5, 15 and 34.

	Wave 5 (N=1032)				Wave 15 (N=993)				Wave 34 (N=1001)			
	Parents of children 0-5 Y.		Adults without children < 18 Y.		Parents of children 0-5 Y.		Adults without children < 18 Y.		Parents of children 0-5 Y.		Adults without children < 18 Y.	
	n/mean	%/SD	n/mean	%/SD	n/mean	%/SD	n/mean	%/SD	n/mean	%/SD	n/mean	%/SD
Group Size	131		744		162	x	698	x	154	x	716	x
**Gender**												
Male	60	45.8 %	364	48.9 %	66	40.7 %	350	50.1 %	79	51.3 %	360	50.3 %
Female	71	54.2 %	380	51.1 %	96	59.3 %	348	49.9 %	75	48.7 %	356	49.7 %
**Age of participant (continuous)**	36,28	10.56	47,95	17.29	36,61	10.01	48,52	16.63	36,36	11.39	46,58	16.54
**Age of participant (categorical)**												
18-29 Y.	31	23.7 %	161	21.6 %	35	21.6 %	136	19.5 %	31	20.1 %	150	20.9 %
30-39 Y.	57	43.5 %	71	9.5 %	83	51.2 %	99	14.2 %	90	58.4 %	121	16.9 %
40-49 Y.	31	23.7 %	125	16.8 %	31	19.1 %	78	11.2 %	18	11.7 %	76	10.6 %
50+ Y.	12	9.2 %	387	52.0 %	13	8.0 %	385	55.2 %	15	9.7 %	369	51.5 %
**Single parent**												
Yes	16	12.2 %	-	-	20	12.3 %	-	-	15	9.7 %	-	-
No	115	87.8 %	-	-	142	87.7 %	-	-	139	90.3 %	-	-
**School education (SE)**												
Up to 9 years of SE	11	8.4 %	84	11.3 %	18	11.1 %	84	12.0 %	14	9.1 %	94	13.1 %
At least 10 years of SE without A-Levels	24	18.3 %	287	38.6 %	41	25.3 %	247	35.4 %	40	26.0 %	225	31.4 %
A-Levels	96	73.3 %	373	50.1 %	103	63.6 %	367	52.6 %	100	64.9 %	397	55.4 %
**Employment**												
Yes	ND	ND	ND	ND	126	77.8 %	422	60.5 %	131	85.1 %	448	62.6 %
No	ND	ND	ND	ND	36	22.2 %	276	39.5 %	23	14.9 %	268	37.4 %
**Health care professional**												
Yes	17	13.0 %	43	5.8 %	16	9.9 %	50	7.2 %	21	13.6 %	59	8.2 %
No	114	87.0 %	701	94.2 %	146	90.1 %	648	92.8 %	133	86.4 %	657	91.8 %
**Systemically relevant profession**												
Yes	ND	ND	ND	ND	ND	ND	ND	ND	58	37.7 %	132	18.4 %
No	ND	ND	ND	ND	ND	ND	ND	ND	96	62.3 %	584	81.6 %
**Net household income (collapsed)**												
<1250€	ND	ND	ND	ND	9	5.6 %	126	18.1 %	5	3.2 %	102	14.2 %
1250-2249€	ND	ND	ND	ND	39	24.1 %	188	26.9 %	34	22.1 %	190	26.5 %
2250-3999€	ND	ND	ND	ND	71	43.8 %	218	31.2 %	73	47.4 %	238	33.2 %
4000+€	ND	ND	ND	ND	34	21.0 %	101	14.5 %	31	20.1 %	128	17.9 %
Missing	ND	ND	ND	ND	9	5.6 %	65	9.3 %	11	7.1 %	658	91.9 %

**Migration background**												
Yes	30	22.9 %	94	12.6 %	40	24.7 %	91	13.0 %	36	23.4 %	135	18.9 %
No	100	76.3 %	648	87.1 %	121	74.7 %	606	86.8 %	118	76.6 %	580	81.0 %
Don't know	1	0.8 %	2	0.3 %	1	0.6 %	1	0.1 %	-	-	1	0.1 %
**Household size**												
Just me	8	6.1 %	249	33.5 %	11	6.8 %	247	35.4 %	12	7.8 %	212	29.6 %
2 people	15	11.5 %	363	48.8 %	21	13.0 %	350	50.1 %	14	9.1 %	378	52.8 %
3-4 people	88	67.2 %	120	16.1 %	108	66.7 %	85	12.2 %	100	64.9 %	108	15.1 %
5 or more	20	15.3 %	12	1.6 %	22	13.6 %	16	2.3 %	26	16.9 %	17	2.4 %
Not answered	0	0%	0	0%	0	0%	0	0%	2	1.3 %	1	0.1 %
**Chronic condition**												
Yes	22	16.8 %	263	35.3 %	38	23.5 %	253	36.2 %	30	19.5 %	259	36.2 %
No	100	76.3 %	454	61.0 %	121	74.7 %	428	61.3 %	120	77.9 %	431	60.2 %
Don't know	9	6.9 %	27	3.6 %	3	1.9 %	17	2.4 %	4	2.6 %	26	3.6 %
**Belonging to risk group**												
Yes	ND	ND	ND	ND	40	24.7 %	416	59.6 %	27	17.5 %	289	40.4 %
No	ND	ND	ND	ND	122	75.3 %	282	40.4 %	117	76.0 %	372	52.0 %
Don't know	ND	ND	ND	ND	0	0.0 %	0	0.0 %	10	6.5 %	55	7.7 %
**Personal COVID-19 infection (5 and 15/34)[a]**												
Yes confirmed / Yes	3	2.3 %	2	0.3 %	2	1.2 %	3	0.4 %	15	9.7 %	32	4.5 %
Yes, not yet confirmed/ No	5	3.8 %	4	0.5 %	4	2.5 %	3	0.4 %	139	90.3 %	684	95.5 %
No / -	102	77.9 %	634	85.2 %	137	84.6 %	638	91.4 %	-	-	-	-
Yes, convalesced / -	ND	ND	ND	ND	1	0.6 %	3	0.4 %	-	-	-	-
Don't know / -	21	16.0 %	104	14.0 %	18	11.1 %	51	7.3 %	-	-	-	-

Supplementary Table 1 –Study sample description of the groups of parents with children aged 0 to 5 years and participants without underage children for waves 5, 15 and 34. SD: Standard deviation. ND: No data available; [a] For this variable the mode of data collection has changed over course of the project (COSMO waves 5 and 15: “Yes, diagnosis confirmed”, “Yes, diagnosis not yet confirmed”, “Yes, convalesced”, “No” and “Don’t know”, wave 34: “Yes”, “No”).

.

S2: Comparison of burden within waves 5, 15 and 34, incl. absolute and relative frequencies as well as Cramer's V and p-values for approximate significance.

Wave 5	Group size (N)	Burden = "Yes" (n)	Burden = "Yes" (%)	Cramer's V	"Approx. Sig."
P. of children 0-5	131	77	58.8%	0.05	0.09
P. Of children 6-17	188	110	58.5%	**0.06**	**0.04**
No children <18	744	368	49.5%	**0.08**	**0.01**
All	1028	533	51.8%		

Wave 15	Group size (N)	Burden = "Yes" (n)	Burden = "Yes" (%)	Cramer's V	"Approx. Sig."
P. of children 0-5	162	58	35.8%	<0.01	0.97
P. Of children 6-17	184	86	46.7%	**0.11**	**<0.01**
No children <18	698	229	32.8%	**0.09**	**<0.01**
All	993	354	35.6%		

Wave 34	Group size (N)	Burden = "Yes" (n)	Burden = "Yes" (%)	Cramer's V	"Approx. Sig."
P. of children 0-5	154	97	63.0%	0.050	0.11
P. Of children 6-17	171	90	52.6%	0.041	0.19
No children <18	716	408	57.0%	0.005	0.87
All	1001	572	57.1%		

Supplementary Table 2 - Cross-sectional comparison of the distribution of burden between the main groups, providing absolute and relative frequencies, Cramer's V and p-values for the approximate significance. Significant results are printed in bold (p<0.05).

S3: Comparison of burden within main groups between waves 5, 15 and 34

	Wave 15 vs. 5		Wave 34 vs. 5		Wave 34 vs. 15	
	OR	95% CI	OR	95% CI	OR	95% CI
P. of children 0-5 Y.	**0.39**	**(0.24-0.63)**	1.19	(0.74-1.92)	**3.05**	**(1.93-4.83)**
P. of children 6-17 Y.	**0.62**	**(0.41-0.94)**	0.79	(0.52-1.20)	1.27	(0.83-1.92)
No children <18 Y.	**0.50**	**(0.40-0.62)**	**1.35**	**(1.10-1.66)**	**2.71**	**(2.19-3.37)**
All	**0.51**	**(0.43-0.62)**	**1.24**	**(1.04-1.48)**	**2.41**	**(2.01-2.88)**

Supplementary Table 3 - Comparison of burden within the main groups between waves 5, 15 and 34, incl. odds ratios (OR) and 95% confidence intervals (95% CI). Significant results are printed in bold (p<0.05).

S4: Multivariate logistic regression for wave 5, 15 and 34

	Wave 5 (March 31st/April 1st 2020)			Wave 15 (June 23rd/24th 2020)			Wave 34 (January 26th/27th 2021)		
	Burden = "Yes" (%)	OR	95% CI	Burden = "Yes" (%)	OR	95% CI	Burden = "Yes" (%)	OR	95% CI
Total	58.5%			46.7%			52.6%		
**Age (continuous)**		1.00	(0.96-1.03)		1.02	(0.98-1.05)		1.00	(0.97-1.03)
**Gender**									
Male (reference)	56.0%	-	-	49.4 %	-	-	45.9 %	-	-
Female	61.4%	1.20	(0.66-2.18)	44.2 %	0.79	(0.44-1.43)	59.3 %	1.70	(0.92-3.16)
**School education**									
Up to 9 years of school education	57.1%	0.90	(0.30-2.82)	38.5 %	0.69	(0.21-2.30)	50.0 %	0.79	(0.25-2.54)
At least 10 years of school education without A-Levels	61.5%	1.17	(0.60-2.28)	50.7 %	1.30	(0.69-2.44)	50.9 %	0.87	(0.44-1.70)
A-Levels (reference)	57.4 %	-	-	45.2 %	-	-	53.9 %	-	-
**Migration background**									
Yes	68.8%	1.72	(0.76-3.91)	61.3 %	**2.34**	**(1.03-5.34)**	69.2 %	2.20	(0.89-5.42)
No/Don't know (reference)	56.8%	-	-	43.8 %	-	-	49.7 %	-	-
**Pseudo-R²**			0.018			0.042			0.050

Supplementary Table 4 - Multivariate logistic regression for burden in different subgroups of parents with school-age children with the covariates age, gender, length of school education and migration background. OR: Odd ratios. 95% CI: 95% confidence interval. Significant results are printed in bold.

S5: Parenthood-related burden in wave 12 and 30

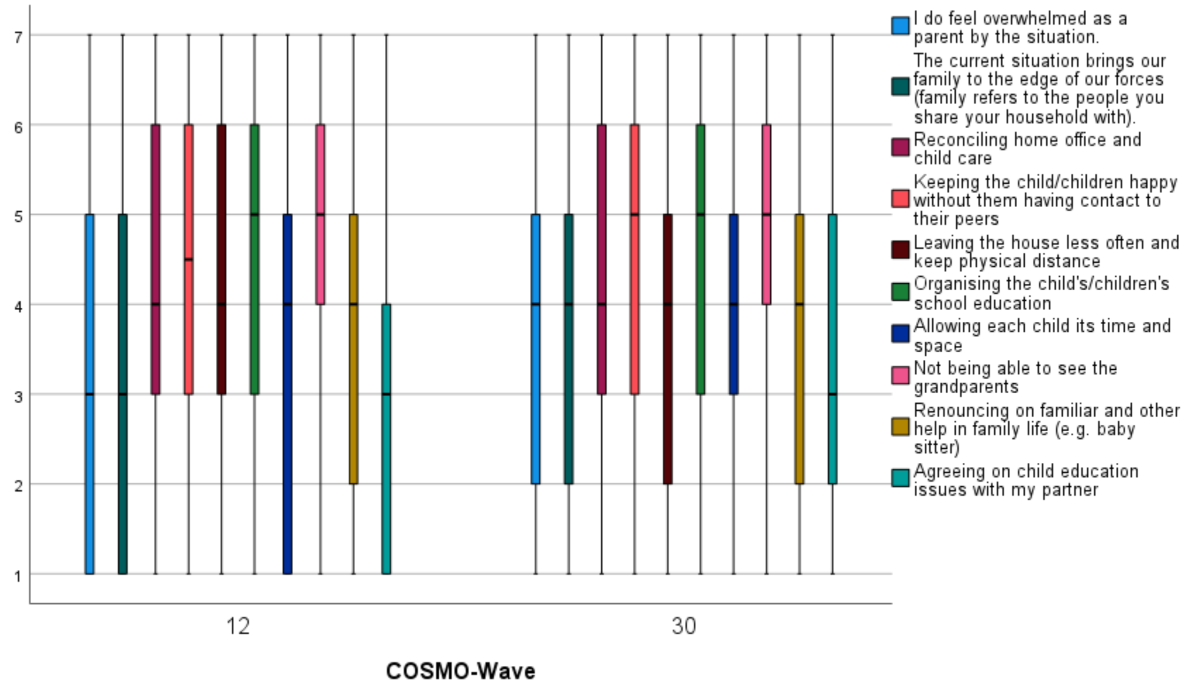

Supplementary Figure 1 - Parenthood-related burden in waves 12 and 30. Boxplot diagrams for the parental burden variable set, comparing parents of school-aged children in wave 12 (May 2020) to wave 30 (December 2020). Response options were 1 "Doesn't apply at all" to 7 "Fully applies" for the first two items, and "Not at all challenging" to 7 "Extremely challenging" for the latter.

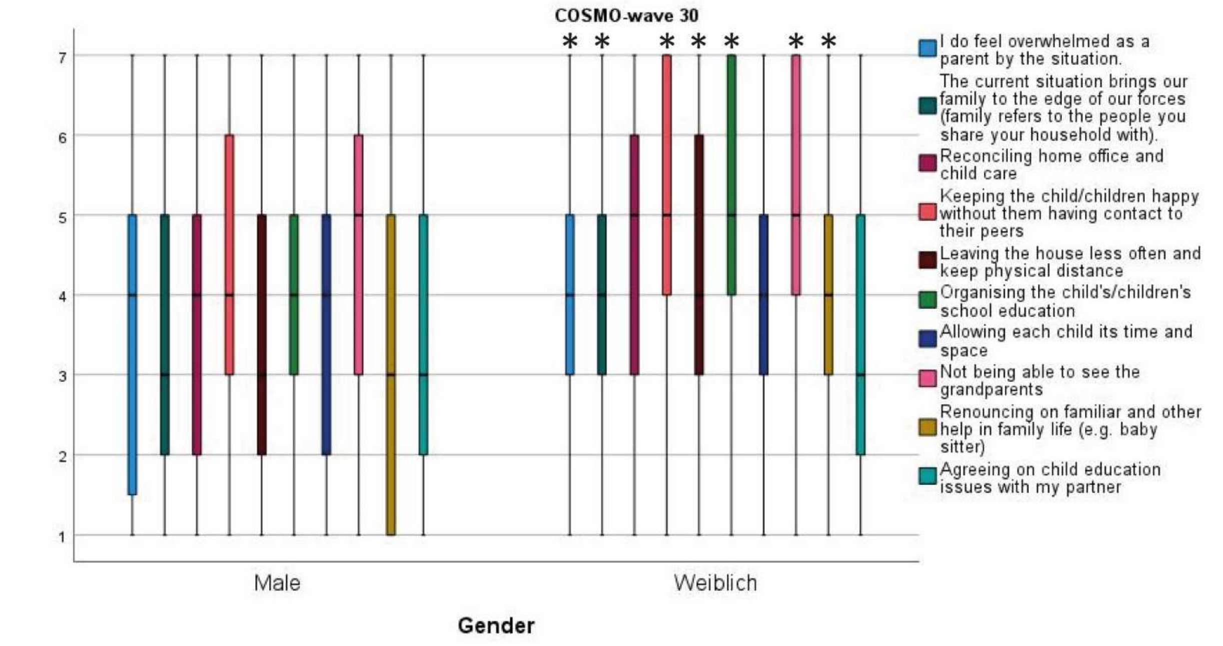

Supplementary Figure 2 - Comparison of parenthood-related burden stratified by gender in wave 30. Boxplot diagrams for the parental burden variable set, comparing mothers to fathers of school-aged children in wave 30 (December 2020). Response options were 1 "Doesn't apply at all" to 7 "Fully applies" for the first two items, and 1 "Not at all challenging" to 7 "Extremely challenging" for the others. Significant results from the Mann-Whitney-U-Test are marked with (*).

S6: Comparison of parenthood-related burden between genders in wave 12 and 30

Wave 12 (19th/20th May 2020)<br>Variable (short name)	Valid Cases (n)	Man-Whitney-U-Test	Z	asymp. Sign.	r (r=Z/n^0,5)
Overwhelmed as parent by the current situation	184	3438,5	-2,229	**0,03**	0,16
Brings our family to the edge of our strengths	184	4126,5	-0,291	0,77	0,02
Reconciling home office and child care	113	1181,5	-2,411	**0,02**	0,23
Keep the children happy under limited contacts with peers	166	2453,0	-3,241	**<0,01**	0,25
Leaving the house less often	167	2826,5	-2,136	**0,03**	0,17
Organising the children's school education	163	2342,0	-3,297	**<0,01**	0,26
Allowing each child its time and space	151	2229,0	-2,349	**0,02**	0,19
Not seeing grandparents anymore	144	1765,5	-3,356	**<0,01**	0,28
Renouncing on additional aid (e.g. baby sitter)	119	1220,5	-2,927	**<0,01**	0,27
Agreeing with partner about children's education	148	2441,5	-1,112	0,27	0,09

Supplementary Table 5 - Comparison of parenthood-related burden between mothers and fathers of school-age children in wave 12 by the Mann-Whitney U test. The number of valid cases varies for the latter eight variables, as not all aspects of parenting apply to all participants. Significant results (p<0.05) are printed in bold.

Wave 30 (15th/16th December 2020)<br>Variable (short name)	Valid Cases (n)	Man-Whitney-U-Test	Z	asymp. Sign.	r
Overwhelmed as parent by the current situation	188	3558	-2.341	**0.02**	0.17
Brings our family to the edge of our strengths	188	3648.5	-2.089	**0.04**	0.15
Reconcile home office and child care	122	1574.5	-1.485	0.14	0.13
Keep the children happy under limited contacts with peers	165	2575	-2.731	**0.01**	0.21
Leaving the house less often	167	2736.5	-2.429	**0.02**	0.19
Organising the children's school education	165	2510.5	-2.951	**<0.01**	0.23
Allowing each child its time and space	161	2792	-1.53	0.13	0.12
Not seeing grandparents anymore	148	2170.5	-2.196	**0.03**	0.18
Renouncing on additional aid (e.g. baby sitter)	120	1411.5	-2.072	**0.04**	0.19
Agreeing with partner about childrens's education	142	2454	-0.257	0.80	0.02

Supplementary Table 6 - Comparison of parenthood-related burden between mothers and fathers of school-age children in wave 30 by the Mann-Whitney U test. The number of valid cases varies for the latter eight variables, as not all aspects of parenting apply to all participants. Significant results (p<0.05) are printed in bold.

6.2 Reference list

1. UNESCO (2020) Education: From disruption to recovery. https://en.unesco.org/covid19/educationresponse. Accessed 28 Sep 2021

2. Fong V, Iarocci G (2020) Child and Family Outcomes Following Pandemics: A Systematic Review and Recommendations on COVID-19 Policies. Journal of pediatric psychology 45:1124–1143. https://doi.org/10.1093/JPEPSY/JSAA092

3. Panda P, Gupta J, Chowdhury S, et al (2021) Psychological and Behavioral Impact of Lockdown and Quarantine Measures for COVID-19 Pandemic on Children, Adolescents and Caregivers: A Systematic Review and Meta-Analysis. Journal of tropical pediatrics 67:. https://doi.org/10.1093/TROPEJ/FMAA122

4. Ravens-Sieberer U, Kaman A, Erhart M, et al (2021) Impact of the COVID-19 pandemic on quality of life and mental health in children and adolescents in Germany. European child & adolescent psychiatry. https://doi.org/10.1007/S00787-021-01726-5

5. Viner R, Russell S, Saulle R, et al (2021) Impacts of school closures on physical and mental health of children and young people: a systematic review. medRxiv 2021.02.10.21251526. https://doi.org/10.1101/2021.02.10.21251526

6. Sprang G, Silman M (2013) Posttraumatic stress disorder in parents and youth after health-related disasters. Disaster medicine and public health preparedness 7:105–110. https://doi.org/10.1017/DMP.2013.22

7. McLaughlin KA, Gadermann AM, Hwang I, et al (2012) Parent psychopathology and offspring mental disorders: results from the WHO World Mental Health Surveys. The British Journal of Psychiatry 200:290. https://doi.org/10.1192/BJP.BP.111.101253

8. Crum KI, Moreland AD (2017) Parental Stress and Children's Social and Behavioral Outcomes: The Role of Abuse Potential over Time. Journal of Child and Family Studies 2017 26:11 26:3067–3078. https://doi.org/10.1007/S10826-017-0822-5

9. Calvano C, Engelke L, di Bella J, et al (2021) Families in the COVID-19 pandemic: parental stress, parent mental health and the occurrence of adverse childhood experiences-results of a representative survey in Germany. European child & adolescent psychiatry. https://doi.org/10.1007/S00787-021-01739-0

10. Rothe J, Buse J, Uhlmann A, et al (2021) Changes in emotions and worries during the Covid-19 pandemic: an online-survey with children and adults with and without mental health conditions. Child and adolescent psychiatry and mental health 15:1. https://doi.org/10.1186/S13034-021-00363-9

11. Huebener M, Waights S, Spiess CK, et al (2021) Parental well-being in times of Covid-19 in Germany. Review of Economics of the Household 19:91–122. https://doi.org/10.1007/S11150-020-09529-4

12. Russell B, Hutchison M, Tambling R, et al (2020) Initial Challenges of Caregiving During COVID-19: Caregiver Burden, Mental Health, and the Parent-Child Relationship. Child psychiatry and human development 51:671–682. https://doi.org/10.1007/S10578-020-01037-X

13. Adams EL, Smith D, Caccavale LJ, Bean MK (2021) Parents Are Stressed! Patterns of Parent Stress Across COVID-19. Frontiers in Psychiatry 12:626456. https://doi.org/10.3389/FPSYT.2021.626456

14. Calarco JM, Anderson E, Meanwell E, Knopf A (2020) “Let’s Not Pretend It’s Fun”: How COVID-19-Related School and Childcare Closures are Damaging Mothers’ Well-Being. https://www.researchgate.net/publication/347216292_Let’s_Not_Pretend_It’s_Fun_How_COVID-19-Related_School_and_Childcare_Closures_are_Damaging_Mothers’_Well-Being. Accessed 28 Sep 2021

15. Cheng Z, Mendolia S, Paloyo A, et al (2021) Working parents, financial insecurity, and childcare: mental health in the time of COVID-19 in the UK. Review of economics of the household 19:123–144. https://doi.org/10.1007/S11150-020-09538-3

16. Marchetti D, Fontanesi L, Mazza C, et al (2020) Parenting-Related Exhaustion During the Italian COVID-19 Lockdown. Journal of pediatric psychology 45:1114–1123. https://doi.org/10.1093/JPEPSY/JSAA093

17. Cusinato M, Iannattone S, Spoto A, et al (2020) Stress, Resilience, and Well-Being in Italian Children and Their Parents during the COVID-19 Pandemic. International journal of environmental research and public health 17:1–17. https://doi.org/10.3390/IJERPH17228297

18. Alonzo D, Popescu M, Ioannides PZ (2021) Mental health impact of the Covid-19 pandemic on parents in high-risk, low income communities. International Journal of Social Psychiatry. https://doi.org/10.1177/0020764021991896

19. Skoda E-M, Spura A, de Bock F, et al (2021) Veränderung der psychischen Belastung in der COVID-19-Pandemie in Deutschland: Ängste, individuelles Verhalten und die Relevanz von Information sowie Vertrauen in Behörden. Bundesgesundheitsblatt - Gesundheitsforschung - Gesundheitsschutz 2021 64:3 64:322–333. https://doi.org/10.1007/S00103-021-03278-0

20. COSMO (2020) Methode | COSMO. https://projekte.uni-erfurt.de/cosmo2020/web/method/. Accessed 28 Sep 2021

21. COSMO (2020) Über das Projekt | COSMO. https://projekte.uni-erfurt.de/cosmo2020/web/about/. Accessed 28 Sep 2021

22. Betsch C, Wieler L, Bosnjak M, et al (2020) Germany COVID-19 Snapshot MOnitoring (COSMO Germany): Monitoring knowledge, risk perceptions, preventive behaviours, and public trust in the current coronavirus outbreak in Germany. https://www.psycharchives.org/handle/20.500.12034/2386. Accessed 28 Sep 2021

23. Schilling J, Tolksdorf K, Marquis A, et al (2021) The different periods of COVID-19 in Germany: a descriptive analysis from January 2020 to February 2021. Bundesgesundheitsblatt - Gesundheitsforschung - Gesundheitsschutz 64:1093–1106. https://doi.org/10.1007/s00103-021-03394-x

24. Maaz K, Diedrich M (2020) Schule unter Pandemiebedingungen: ‘Lockdown’ – ‘Hybridmodell’ – ‘Normalbetrieb’| APuZ. In: Bundeszentrale für politische Bildung. https://www.bpb.de/apuz/schule-2020/322683/schule-unter-pandemiebedingungen. Accessed 28 Sep 2021

25. das Deutsche Schulportal (2020) Bundesländer - Fahrplan für die Schulöffnungen - Das haben die Länderchefs entschieden. https://deutsches-schulportal.de/bildungswesen/schuloeffnung-das-haben-die-laenderchefs-entschieden/. Accessed 28 Sep 2021

26. (2020) Schulferien Deutschland 2020. https://www.schulferien.org/deutschland/ferien/2020/. Accessed 28 Sep 2021

27. Hesse M (2020) Bundesweiter Lockdown Regelungen Schulen und Kitas. Kultusminsterium Baden-Württemberg

28. Arbeitsgemeinschaft der Wissenschaftlichen Medizinischen Fachgesellschaften (2021) S3-Leitlinie Maßnahmen zur Prävention und Kontrolle der SARS-CoV-2-Übertragung in Schulen | Lebende Leitlinie Kurzfassung Hinweise zur Kurzfassung

29. Thorell L, Skoglund C, de la Peña. AG, et al (2021) Parental experiences of homeschooling during the COVID-19 pandemic: differences between seven European countries and between children with and without mental health conditions. European child & adolescent psychiatry. https://doi.org/10.1007/S00787-020-01706-1

30. Hipp L, Bünning M (2020) Parenthood as a driver of increased gender inequality during COVID-19? Exploratory evidence from Germany. European Societies 23:S658–S673. https://doi.org/10.1080/14616696.2020.1833229

31. Mohler-Kuo M, Dzemaili S, Foster S, et al (2021) Stress and Mental Health among Children/Adolescents, Their Parents, and Young Adults during the First COVID-19 Lockdown in Switzerland. International Journal of Environmental Research and Public Health 18:4668. https://doi.org/10.3390/ijerph18094668

32. Kreyenfeld M, Zinn S (2021) Coronavirus and care: How the coronavirus crisis affected fathers' involvement in Germany. DEMOGRAPHIC RESEARCH 44:. https://doi.org/10.4054/DemRes.2021.44.4

33. Czymara CS, Langenkamp A, Cano T (2021) Cause for concerns: gender inequality in experiencing the COVID-19 lockdown in Germany. European Societies 23:S68–S81. https://doi.org/10.1080/14616696.2020.1808692

34. Collins C, Landivar LC, Ruppanner L, Scarborough WJ (2021) COVID-19 and the gender gap in work hours. Gender, Work & Organization 28:101–112. https://doi.org/10.1111/GWAO.12506

35. Andrew A, Cattan S, Dias MC, et al (2020) The Gendered Division of Paid and Domestic Work under Lockdown. https://ssrn.com/abstract=3654937.

36. Andrew A, Cattan S, Dias MC, et al (2020) How are mothers and fathers balancing work and family under lockdown? https://doi.org/10.1920/BN.IFS.2020.BN0290

37. WHO/Europe (2020) Strengthening and adjusting public health measures throughout the COVID-19 transition phases. Policy considerations for the WHO European Region, 24 April 2020

38. COVID-19 Dashboard Germany. In: RKI. https://experience.arcgis.com/experience/478220a4c454480e823b17327b2bf1d4%20. Accessed 31 May 2021.
